# Evaluating contributions of progressive ratio analysis to economic metrics of demand

**DOI:** 10.1002/jeab.70077

**Published:** 2025-12-26

**Authors:** Joseph M. Lambert, Maria A. Osina, Johanna L. Staubitz, Derek D. Reed, Gregory J. Madden

**Affiliations:** ^1^ Department of Special Education Vanderbilt University Nashville TN USA; ^2^ Department of Psychology and Human Development Vanderbilt University Nashville TN USA; ^3^ Institutes for Behavior Resources, Inc. Baltimore MD USA; ^4^ Department of Psychology Utah State University Logan UT USA

**Keywords:** behavioral economics, breakpoint, demand elasticity, prospective controlled consecutive case series, relative reinforcer efficacy

## Abstract

Progressive ratio analysis (PRA) has been used to quantify the relative reinforcer efficacy of various programmed consequences across basic and applied research paradigms. It has also been used as an alternative methodology for demand‐curve analysis. In this study, we enrolled 96 consenting adults with disabilities to participate in a translational controlled consecutive case series. Specifically, we compensated participants for using an arbitrary response (e.g., a die roll) to demonstrate the circumstances under which they would work to earn preferred reinforcers in both Basis *x* PRA and progressive fixed ratio analysis (PFRA) paradigms. Using *t* tests of logarithmically transformed Pearson correlation coefficients, we established that Basis *x* PRA did not correlate with metrics of demand elasticity obtained from PFRA. However, Basis *x* PRA significantly predicted multiple metrics of equilibrium observed during PFRA. Consequently, the assessment likely retains prescriptive value across a number of domains.

Various distinct procedures have carried the label *progressive ratio* analysis (PRA). The variable that unites methodologies under this moniker is that all include response requirements for reinforcement that increase over time (e.g., Baron et al., 1992; Hodos & Kalman, 1963; Johnson & Bickel, 2006). Jarmolowicz and Lattal (2010) recently detailed similarities and differences across common arrangements. They also highlighted reasons, based on logic and evidence, for why it could be problematic to conflate distinctive methodological elements and associated interpretations. After reviewing the literature for prevalence and precedent, these authors outlined three classes of methodology and proposed new labels for each.

The first, *PRA*, retains the original name because it is the oldest and most frequently employed strategy. A PRA describes any procedure in which response requirements are increased within session following each successive reinforcer delivery. In this preparation, session duration varies as a function of response output and sessions are terminated after responding has ceased for a specified period. *Progressive fixed ratio* analysis (PFRA) represents a second class of procedures for which response requirements increase between sessions instead of within sessions. Finally, Basis *x* schedules are similar to PRA and PFRA in that response requirements increase across time as a function of either component schedule (Basis *x* PRA) or session (Basis *x* PFRA) completion. However, unlike PRA and PFRA, response requirements are increased intermittently. That is, in Basis *x* schedules, *x* refers to the number of ratios or sessions that are completed at each successive response requirement. For example, in a Basis 2 PRA3, the ratio requirement increases by three following every two reinforcer deliveries.

## Demand‐curve analysis

With an acknowledgement that a given reinforcer can support different amounts of behavior at different unit prices (e.g., Borrero et al., 2007), behavioral economists will often conduct parametric assessments referred to as demand‐curve analysis. The objective of demand‐curve analysis is to quantify equilibrium points between supply and demand (i.e., the net response and reinforcer outputs maintained by a given reinforcer at a given unit price). After establishing equilibrium at various systematically arranged unit prices, researchers then quantify the reinforcer's *essential value*—a value derived from the amount of behavior a given reinforcer will support in the face of increasing costs (Gilroy, 2023).

In these experiments, session durations are typically long to ensure that the only constraint on reinforcer consumption is the price of the reinforcer (Hursh, 1980). Prices are also typically changed between sessions (e.g., Bickel & Madden, 1999) in either ascending or randomized order (e.g., Giordano et al., 2001). Using the taxonomy proposed by Jarmolowicz & Lattal (2010), it would be appropriate to label the methods of most demand‐curve analyses as Basis *x* PFRAs.

All else being equal, reinforcer consumption is highest when unit prices (ratio schedule) are lowest. Consequently, the quantity of reinforcer consumption (i.e., *Q*) that occurs in the absence of economic constraint (sometimes also referred to as *Q*_0_, bliss‐point consumption, or demand intensity) is a variable of interest to behavioral economists because it represents a consumption threshold beyond which access to a commodity no longer functions as a reinforcer. For example, if a child would only eat seven M&Ms if given unconstrained access to them, then it is highly unlikely that the contingent delivery of an 8th M&M would have a reinforcing effect on their behavior (for a discussion of the applied implications of *diminishing marginal utility*, see Madden et al., 2024).

When unit prices are increased during demand‐curve analysis, individuals must increase response output to “defend” (maintain) previous consumption patterns (e.g., *Q*_0_). The extent to which an individual does so varies considerably and often depends on the nature of the economic system (i.e., open vs. closed economy), qualitative features of the commodity in question (e.g., essential/nonessential), and the existence and nature of alternative sources of reinforcement (e.g., complements/substitutes).

**FIGURE 1:**
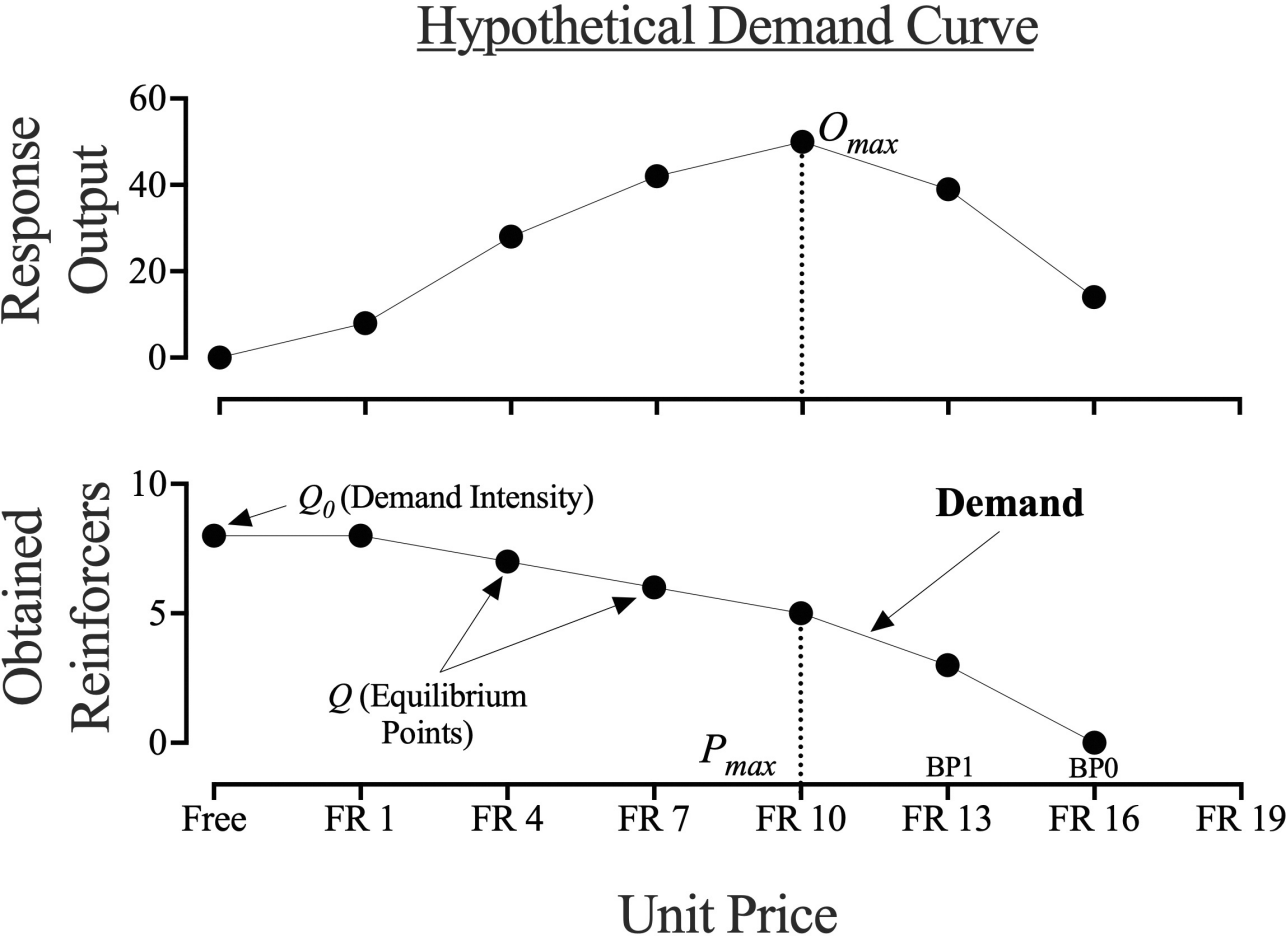
Hypothetical demand curve demonstrating various economic value metrics. See text for definitions. FR = fixed ratio.

When the ratio of change to consumption is less than the ratio of change to unit price (i.e., when price increases lead to increases in overall responding), demand is considered *inelastic*. When the inverse is true, demand is considered *elastic* (Gilroy, 2020). The unit price that quantifies the change in demand from inelastic to elastic is referred to as *P*_*max*_ and is important because it identifies the price that supports the highest equilibrium point, or greatest amount of responding (i.e., *O*_*max*_; see Figure 1).

A summary (across price) metric of the *essential value* of the reinforcer entails a description of an interaction between equilibrium and elasticity and is given by *EV* = *Q*_0_ × *P*_*max*_ (Gilroy, 2023). A reinforcer with a large essential value is one that the individual works hard to acquire in the face of price increases; small essential values mean that price increases are met with weak efforts to continue consuming the reinforcer. Economic metrics such as *P*_*max*_ vary across commodities and are important because they serve as an index of value that can, among other things, be used to optimize the value of reinforcers earmarked for instructional programming (e.g., Gilroy et al., 2018), design treatments for challenging behavior (e.g., Gilroy et al., 2019; Roane et al., 2007), and predict the relative influence of baseline reinforcement conditions on bursting and persistence during extinction (e.g., Lambert et al., 2024).

## Attempts to improve the efficiency of demand‐curve analysis

Despite the potential benefits of demand‐curve analysis, Basis *x* PFRA is prohibitively time consuming (Leon et al., 2021). Consequently, some researchers have explored the validity of alternative methods for obtaining key economic metrics of demand elasticity (e.g., *P*_*max*_). For example, although not feasible for all populations (e.g., those with limited verbal repertoires), hypothetical purchasing tasks are efficient and can circumvent many concerns associated with progressive contingencies (e.g., Murphy & MacKillop, 2006). Rapid demand‐curve assays that leverage PFRA can also serve as a viable alternative to Basis *x* PFRA and may be better suited than hypothetical purchasing tasks for intervention paradigms involving individuals with disabilities because it does not hold verbal comprehension as a prerequisite and does generate data that fit well with existing models of demand (Madden et al., 2005; Raslear et al., 1988). However, purportedly because PFRA is time consuming (despite being more efficient than Basis *x* PFRA), this methodology has yet to be leveraged in research targeting applied populations.

Under an assumption that data obtained from PFRA and PRA can be interpreted in similar ways, some researchers have conducted demand‐curve analyses using PRA (e.g., Leon et al., 2021) or Basis *x* PRA (e.g., Gilroy et al., 2021; Reed et al., 2009). For example, Reed et al. (2009) exposed participants to three Basis *x* PRA tests. They then segmented response and reinforcer data from each session according to the specific unit prices that produced them, collapsed segmented data from all three sessions into unit‐price‐specific bins, and then identified *P*_*max*_ by entering these collapsed and segmented outputs into the quantitative model of demand described by Hursh et al. (1988).

## Exploring the validity of economic metrics derived from PRA

Notably, in PFRA and Basis *x* PFRA, the only within‐session contingency programmed to operate on behavior stems from the component ratio requirement under analysis (e.g., FR 4). That is, responding produces reinforcement at the specified ratio requirement for as long as a participant chooses to respond (e.g., Griffiths et al., 1975; Johnson & Bickel, 2006). For contrast, both PRA and Basis *x* PRA introduce a second contingency that operates on behavior independently of component ratio requirements. That is, responding produces reinforcement while simultaneously contributing to subsequent increases in response effort (analogous to a game of diminishing returns). To the extent that participants are sensitive to these contingent increases, PRA and Basis *x* PRA can contribute to performance patterns that function to reset ratio requirements (Hurwitz & Harzem, 1968) and/or expedite session termination (Dardano, 1973, 1974).

Consequently, and unlike PFRA and *Basis x* PFRA, price‐specific consumption patterns in PRA and *Basis x* PRA cannot reflect an equilibrium between supply and demand because these arrangements (1) impose an artificial ceiling on reinforcer consumption at all given unit prices, (2) ensure that responding at higher unit prices is differentially affected by satiation (i.e., *Basis x* consumption requirements at all lower unit prices are always satisfied prior to the introduction of higher unit prices, within a single session), and (3) include a potentially suppressive responsive contingency not accounted for by the logic that informs demand‐curve analysis.

Relevant to this point, although Reed et al. (2009) successfully established ordinal hierarchies of efficacy using Basis *x* PRA, minimal empirical work has been done to evaluate the validity of deriving economic metrics from Basis *x* PRA. In fact, both basic and applied researchers have offered compelling preliminary evidence to suggest that *P*_*max*_ derived from PRA or Basis *x* PRA is unlikely to correspond to the values obtained by their PFRA and Basis *x* PFRA counterparts (e.g., Foster et al., 1997; Leon et al., 2021; Tan & Hackenberg, 2015). Notwithstanding, it may be that predictions of *P*_*max*_ made by PRA or Basis *x* PRA are reliability skewed (e.g., consistently overestimated; cf. Foster et al., 1997)—potentially giving them a degree of predictive utility when demand‐curve analysis is not otherwise possible. However, small sample sizes and procedural asymmetries across studies have precluded our ability to determine the extent to which there are correlations between the results from these methodologies (Roane, 2008).

More promising, predictive relations between PRA‐derived breakpoints and measures of equilibrium have been established by various researchers (e.g., Johnson & Bickel, 2006; Madden et al., 2007a, 2007b; Rodefer & Carroll, 1997). Although such correlations have not been established for individuals with disabilities, the relation is still noteworthy given that measures of equilibrium offer clear advantages to intervention design that are distinct from those offered by *P*_*max*_ (e.g., Acuff et al., 2024; Martínez‐Loredo et al., 2021) and given that there is already a published precedent for imbedding PRA and Basis *x* PRA into applied research paradigms.

For context, the degree to which responding persists in PRA and Basis *x* PRA has historically been used to quantify a reinforcer's value (e.g., Hodos, 1961; Tustin, 1994). That is, by noting the last schedule requirement that yields at least one reinforcer (breakpoint [BP] 1) or the first schedule requirement that does not yield reinforcement (BP0), researchers have compared breakpoints produced by various reinforcers inserted into PRA or Basis *x* PRA and ranked their relative value in ways analogous to the rankings generated by preference assessments.

In the applied literature, this strategy has been used to establish the relative value of contingent access to various social interactions (Jerome & Sturmey, 2008, 2014) and to low‐preferred stimuli earmarked for instructional programming (Francisco et al., 2008; Glover et al., 2008; Penrod et al., 2008). Breakpoints obtained from PRA have also been used to quantify the relative influence of systematic manipulations to reinforcer magnitude (e.g., Trosclair‐Lasserre et al., 2008), to inform reinforcer selection for treatments of challenging behavior (Roane et al., 2001), and to quantify and counteract response bias during treatments of challenging behavior (e.g., DeLeon et al., 2000).

## Purpose

As there is uncertainty about whether or how Basis *x* PRAs might be used to predict either the elasticity (*P*_*max*_) or equilibrium (demand intensity, *O*_*max*_) of reinforcer demand for individuals with disabilities, large‐*N* research capable of exploring the topic offers clear value to future translational and applied research efforts. Thus, holding participant, reinforcer, and parameters of progressive contingencies (e.g., step size, step type) constant, the purpose of this study was to evaluate whether data obtained from Basis *x* PRA correlated with economic metrics of demand produced by PFRA.

## METHOD

This study was conducted in conjunction with a randomized control trial designed to explore the functionality of considering the economic concepts of elasticity and diminishing marginal utility when attempting to predict and control extinction bursts in adults with disabilities (Lambert et al., 2024). Thus, a subset of our assessment results (i.e., Basis *x* PRA and PFRA data for 69 participants) are available as supplemental materials for that Lambert et al. (2024).

### Participants

Across a 2‐year period, we recruited and consented 111 adults with disabilities to participate in this study. Individuals were eligible if they (a) were older than 18, (b) had a disability diagnosis under the Individuals with Disabilities Education Act (IDEA, 2004), (c) could exhibit the ability to physically interact with study materials in a manner consistent with the study's requirements (e.g., exhibiting sufficient motor skills to squeeze a clothespin), and (d) consented to participate prior to study onset. If participants could not legally consent, we obtained consent from guardians, and assent from participants was obtained before each session. Of the 111 participants enrolled, 96 completed the study (13.5% attrition). Reasons for attrition included scheduling conflicts, health concerns unrelated to this study, and loss of interest. The average participant age was 40 years (range 18–79), with a median of 34 years. Participant demographic information and appointment details are displayed in the supplemental materials.

### Recruitment, consent, and settings

Recruitment materials were disseminated through seven organizations serving adults with disabilities across two states. Of the four organizations with interested clients, three specialized in residential and day‐service programming and one specialized in inclusive postsecondary education in a university setting. All recruitment efforts were mediated by third party representatives unaffiliated with the study who had a professionally established investment in the well‐being of prospective participants (e.g., program directors). That is, researchers never made initial contact with prospective participants and only contacted those whose interests had been vetted and approved by said mediators.

When interest was expressed, researchers scheduled a 30–45 min meeting with each prospective participant (and/or their conservator). During this meeting, researchers explained that they were interested in learning how prospective participants “valued” reinforcers and that this could be learned by identifying the circumstances under which participants chose to stay at a table and work for a reinforcer selected by them. We clarified that participants would never be forced to participate and that our findings would not be valid if participants ever felt compelled to work for any reason other than to obtain the selected reinforcer. After answering all residual study‐related questions (e.g., study goals, study methods, compensation for participation), we initiated the informed consent process. Because study activities were not directly beneficial to participants, we scheduled appointments only at times not committed to work, instructional programming, and/or preferred leisure activities.

Sessions were conducted at the organizations from which participants were recruited (day program, workshop, or university settings for 69.8% (67 of 96) of participants and home settings for 30.2% (29 of 96) of participants). These organizations maintained their business‐as‐usual staffing ratios and protocols, monitored study appointments (when needed), and maintained responsibility for all behavioral programming (when relevant). Sessions were conducted in a convenient location in these settings, with the participant seated at a table in an area visually accessible to endogenous stakeholders (e.g., staff, parents).

### Study materials

Materials used for the target response included a die or a clothespin. Edible reinforcers were placed on plates, and audio reinforcers were presented through a sound system. Data were collected with handheld computers using Countee software. Video cameras were sometimes used to record behavioral data when a secondary observer was unavailable. Data were stored on a password protected and encrypted HIPAA‐compliant cloud‐based server and were deleted after reliability data were obtained.

Participant‐specific binders included relevant data collection (e.g., procedural fidelity sheets) and summary materials for all phases of the study. Binders also included a sign‐in sheet and punch card that researchers used at the end of each appointment to help participants track when they could expect to receive each $25 gift card (described below).

### Target response definitions, data recording, and dependent variables

Target responses were intentionally arbitrary to decrease the probability that response–reinforcer relations between them and programmed consequences had been established prior to study onset. For 93 participants, target responding entailed die rolling (i.e., picking up a die and putting [or dropping] it back down). For the remaining three participants, target responding entailed manipulating a clothespin (i.e., picking up a clothespin, squeezing it, and putting it back down). Trained observers collected continuous timed‐event measures of the frequency of these target responses. They also recorded the FR response requirements for each reinforcer, reinforcer delivery event, and overall session duration. Reinforcer delivery entailed either placing an edible item on a plate situated in front of participants (when relevant) or turning on a preferred song for 30 s (when relevant). Reinforcer unit sizes (e.g., the size of the morsel, the duration of audio exposure) were standardized to ensure the relevance of the economic concept of “unit price” to study results and were sufficiently small to decrease the probability of satiation following consumption of a single unit. All reinforcers were selected based on verbal reports of preference and novelty (i.e., we tried to select preferred reinforcers that participants did not typically have access to).

### Interobserver agreement

We calculated IOA for each dependent variable by comparing frequency counts scored by primary and secondary observers, specifically, by dividing the smaller count by the larger count and multiplying by 100 (e.g., Madden et al., 2021). We then calculated a session agreement by aggregating IOA scores for each dependent variable. Across participants, average IOA for Basis *x* PRAs was 99.4% (range 93%–100%, median = 100%, mode = 100%) and was calculated for 33.4% (193 of 578) of sessions. Average IOA for PFRAs was 99% (range 84.6%–100%, median = 100%, mode = 100%) and was calculated for 42.1% (185 of 439) of sessions. Participant‐specific IOA data are displayed in the Supporting Information.

### Procedural fidelity

Implementer fidelity evaluations entailed responses to yes/no checklists that described critical elements of session implementation. When a researcher implemented a session element as described, observers scored a “yes.” When they did not, observers scored a “no.” Session fidelity was then calculated by dividing all yes responses by the sum of yes + no responses and multiplying by 100 (for sample fidelity sheets, see “appointment mechanics” in the Supplemental Materials). Across participants, the mean fidelity score for Basis *x* PRAs was 99.6% (range 93.8%–100%, median = 100%, mode = 100%) and was calculated for 94.3% (545 of 578) of sessions. The mean fidelity score for PFRAs was 99.8% (range 95.2%–100%, median = 100%, mode = 100%) and was calculated for 91.6% (402 of 439) of sessions. Participant‐specific fidelity data are displayed in the Supporting Information.

### Experimental design

We used reversal designs to demonstrate that target responding was controlled by programmed consequences for all participants. To explore the criterion‐related validity of the economic predictions made by Basis *x* PRAs, we conducted a prospective controlled consecutive case series (CCCS; Hagopian, 2020). As case‐series logic calls for researchers to report all data produced by consecutively enrolled participants who have been exposed to the same procedures (regardless of outcome), this design arrangement allowed us to address not only the possibility of a specified outcome (e.g., correspondence between predicted and observed *O*_*max*_ and *P*_*max*_ for a given participant) but also its prevalence.

Each participant completed a Basis *x* PRA and a PFRA, in that order. This sequence was selected because Basis *x* PRA outcomes served as the prerandomization matching variable in Lambert et al. (2024). The sequence was also selected because the Basis *x* PRA described by Reed et al. (2009)—our model for Basis *x* PRA design—called for three distinct exposures to their test condition. Thus, it was easy and convenient to intersperse control sessions into the Basis *x* PRA to efficiently establish experimental control of programmed consequences over target responding (via rapid iterative alternation; Bailey et al., 2024; Ledford et al., 2024). In the cases for which control was not established, this sequence allowed us to take immediate corrective action (e.g., change the response, change the environment) before exposing participants to the assessment we anticipated would be more time and resource intensive (i.e., PFRA). Because neither the response nor the reinforcer changed when participants progressed to PFRA, we did not attempt to reestablish control of programmed reinforcers over target responses during this second assessment.

Because participant characteristics, parametric differences in how schedule requirements are increased (e.g., arithmetic vs. geometric progressions), amount and quality of reinforcers delivered, session‐termination criteria, and complexity and effort of the target response can all differentially affect assessment outcomes (Kaplan et al., 2018; Jarmolowicz & Lattal, 2010; Roane, 2008), we held these variables constant across both assessments. To ensure assessment sensitivity, we kept step sizes small and unit price progression occurred in ascending order (cf. Kaplan et al., 2018). Because PRAs can sometimes be experienced as aversive by participants (Leon et al., 2021; Poling, 2010), we attempted to attenuate any aversive qualities of our Basis *x* PRA and PFRA assessment procedures by clearly and frequently emphasizing their optional nature and by ensuring that alternative reinforcers (e.g., access to other items, researcher attention) were always available for low‐effort alternative response options (cf. Leon et al., 2021).

Importantly, there was one planned procedural asymmetry across assessments. Specifically, methods described by Reed et al. (2009) called for Basis *x* PRAs to be conducted three times each (purportedly to accumulate enough data to use the quantitative model described by Hursh et al., 1988). Whereas we followed this guidance for our own Basis *x* PRAs, limited fiscal and human resources precluded our ability to conduct such replications for our PFRA comparisons. Notwithstanding, because rapid demand‐curve assays that leverage PFRA retain their content validity (e.g., Madden et al., 2005; Raslear et al., 1988) and ensure unconstrained within‐session access to reinforcement at each schedule value, they offered a direct and valid (albeit less conservative) test of the predictions made by each participant's Basis *x* PRA results.

### Compensation

In addition to programmed reinforcers, all participants were compensated with gift cards at a rate commensurate with minimum wage in the states in which this study was conducted (i.e., $7.25/hr). To accomplish this, each participant was given a 10‐hole “punch card” (stored by researchers between appointments). At the end of every appointment, researchers punched a hole in (or initialed) the card for every 20 min that the participant spent in the appointment. When all 10 holes had been initialed or punched, researchers immediately delivered a $25 gift card and initiated a new punch card during the next appointment.

Importantly, researchers aways rounded up. For example, a participant who terminated an appointment after a 60‐s session would have earned one punch. A participant who terminated an appointment after 20 min and 60 s would have earned two punches. When participants could not manage their own money, gift cards were given to their conservators with the promise that the money would be spent to satisfy participants' preferences and/or needs.

### Procedures

For each participant, we scheduled between one and five appointments per day across 1–5 days per week. To the extent possible, we held appointments at the same times each day and week to enhance consistency across motivating operations and other setting events. Similarly, we attempted to offer reinforcer options that were not typically available to participants (e.g., preferred snacks or activities not found in their homes). However, as it would have been a rights violation to ask caretakers to withhold preferred items or activities outside of formal study sessions, we never made this request. Thus, although we took steps to establish a closed economy, we could not guarantee it.

All participants completed the same progression: (1) interview (i.e., informed consent, appointment scheduling, identification of programmed reinforcers and target responses), (2) target‐response training, (3) assessment of demand intensity (*Q*_0_), (3) Basis *x* PRA, (4) PFRA. To minimize unintended coercive practice, all assessment‐related activities were framed as a choice. Specifically, researchers initiated each appointment with a 5‐min casual conversation. Prior to each session, researchers displayed the apparatus for the target response (e.g., dice) and a low‐preferred alternative activity (e.g., a magazine or a toy) and paraphrased a script unique to each study condition (described below). The specific language for each script was shaped by problem‐solving efforts during pilot‐study initiatives as an attempt to preempt the generation of inaccurate self‐rules about prevailing contingencies and/or researcher expectations. All appointments terminated the moment a participant indicated they wanted it to end (see “appointment mechanics” in the Supporting Information).

#### Response training

All target responses entailed a multistep process (e.g., pick up a clothespin, squeeze it, put it down). The final step of each response (e.g., putting down a clothespin) automatically set up another opportunity to initiate it (e.g., picking up the clothespin). The target responses (die rolling or clothespin squeezing) were easy to execute and did not occur in the absence of our programmed consequences.

The target response topography was matched to each participant's skill set. During training, researchers modeled the correct response and instructed participants to do the same. If they could not, researchers used a least‐to‐most prompting procedure to assist initial responding (e.g., gestures, manual guidance) and then delivered praise after each completion (Collins, 2022). If participants still could not independently emit the response, they were trained in the other response topography.

#### Assessment of demand intensity

Demand intensity (*Q*_0_) was assessed once. Facing an array of potential reinforcers, participants were asked to pick one that they would consume for the remainder of the study. The available options were in accord with participants' stated preferences and dietary restrictions. When the participant made their selection, researchers paraphrased the following statement:

*We'll start working for (reinforcer) the next time I come. Right now, I'm just going to let you have as much of it as you want. Each time you take one and eat it [listen to it], I'll replace it with another. When you don't want to eat [listen] anymore, don't take anymore. Remember, you can always choose to play with this (low‐preferred alternative) or tell me that you're done or that you'd rather talk to me than eat [listen]*.

For participants with complex communication needs, contingency reviews were simplified and experience based (i.e., responses were prompted with gestures and/or manual guidance and were consequated in conjunction with the delivery of key instructions).

Edible and auditory reinforcers were selected based on verbal report of participant preference and novelty (i.e., we tried to select reinforcers that participants did not typically have access to). For participants who earned edible reinforcers, researchers placed carefully measured and equal‐sized units of each food on a plate in front of the participant one at a time (after they made a choice and were given free access to their favorite). Each time participants picked up a morsel to consume, another was placed on the plate. Participants also had access to low‐preferred alternative activities intended to compete with reinforcer consumption as the value of consumption depleted. Likewise, researchers did not speak to participants during sessions. If participants attempted to initiate a conversation while also consuming reinforcement, researchers indicated that they could not talk while participants were eating/listening to music but that they *could* talk if participants decided to terminate the session.

Sessions terminated when a participant refrained from consuming a reinforcer for 60 s (e.g., because they began to engage with the alternative activity), stated that they wished to terminate the session, touched a laminated “stop” card, or after 1 hr had elapsed (no assessment was ever terminated due to the passage of time). For participants with complex communication needs, we also accepted the act of walking away from the table as an indication of dissent/session termination.

For participants with dietary restrictions, programmed consequences were auditory and reinforcement procedures were adapted from Lambert et al. (2019). That is, researchers began the session by playing the identified song on an iPhone. If the song finished before participants terminated a session, researchers allowed the next song of the relevant album to begin. Thus, participants could listen to up to an hour of music from the same album that contained the identified song. After session termination, we divided session duration by 30 to determine the number of 30‐s units each participant had consumed during the assessment. To avoid confounds associated with satiation, no additional sessions were conducted during the same appointment in which this condition was conducted.

#### Basis *x* PRA

This assessment was adapted from procedures described by Reed et al. (2009). During the assessment, participants were exposed to three test sessions, each interspersed with control sessions. Controls occurred prior to tests. To avoid satiation, only one test was conducted per appointment. Control and test sessions of the Basis *x* PRA were similar to the assessment of demand intensity, with the following modifications.

##### Control

Researchers placed the target‐response apparatus (e.g., dice) in front of participants and delivered a paraphrase of the following contingency review:

*We are going to start a session now. This just means I'm going to give you an opportunity to (task). When you do this (model task for participant), nothing is going to happen, so only do it if you like doing it. If it's boring, don't do it. During sessions, I cannot talk to you. That's just a rule I have to follow. It doesn't mean I don't want to talk or that I'm not happy. If you don't want to wait or would rather talk to me, you only need to stop (target response) for one minute. You can also just say, “I'm done” or point to this stop card. While you wait, you can (alternative activity) if you like*.

Researchers then began a session by saying, “3, 2, 1, start” and looking down to avoid eye contact. If participants emitted a target response during a control session, researchers interrupted additional responding and paraphrased the following statement:

*Remember, right now I just need to know that you know that responding will not get you anything. The way that I'll know that is when you stop responding for one minute. While you wait, you can play with (low‐preferred alternative). Or, if you're bored and don't want to wait anymore, you can say “all done” and we'll just end the session*.

They then reset their timer and restarted the session. This continued until researchers obtained a 60‐s sample of the absence of target responding or participants asked to stop the session. We determined that both cases represented a disinclination to continue to emit the response in the absence of programmed consequences and provided compelling evidence that responding that occurred during subsequently conducted test sessions was maintained by programmed consequences.

##### Test

Target responding during test sessions was exposed to a Basis 2 PR3 schedule. Specifically, after every other reinforcer delivery, price increased by three responses (i.e., FR 1, FR 1, FR 4, FR 4, FR 7, FR 7, etc.). At the beginning of each session, researchers paraphrased the following statement:

*We are going to start a session now. This just means I'm going to give you an opportunity to earn (reinforcer). At first it will be easy to earn (reinforcer), then it will take more time. During sessions, I cannot talk to you. That's just a rule I have to follow. It doesn't mean I don't want to talk or that I'm not happy. If you want (reinforcer) after the session starts, you can work on (target response). If you stop wanting (reinforcer), get too bored doing (task), or just want to talk to me, you only need to stop (target response) for one minute. You can also just say, “I'm done” or point to this stop card. While you wait, you can (alternative activity) if you like*.

Researchers then began a session by saying, “3, 2, 1, start” and looking down to avoid eye contact. If participants asked how many responses were needed to earn the next reinforcer, the researcher told them. For those who needed it (e.g., participants who received this type of support under typical circumstances), the researcher also counted out loud the number of responses made each time a new response was made. This was typically faded after delivery of the first 3 to 5 reinforcers of a given session.

Approximately once every 5 min, researchers reminded participants that they should not respond to make the researcher happy; that they should only continue responding if they wanted the programmed reinforcer; and that they should stop the moment they were tired of responding, didn't want the reinforcer, or simply wanted to quit. Sessions terminated when a participant refrained from emitting a target response for 60 s, stated that they wished to terminate the session, touched a laminated “stop” card, walked away from the table, or after 1 hr had elapsed (no session was ever terminated due to the passage of time).

#### PFRA

This assessment was adapted from procedures described by Madden et al. (2005) and was similar to the Basis *x* PRA with a few exceptions. First, the PFRA did not include a control condition. Next, schedule values were fixed (held constant) for the entire session. That is, response requirements increased by three across sessions rather than across reinforcer deliveries (this modification allowed us to establish equilibrium points for programmed reinforcers at each schedule value). To control for satiation, we conducted one PFRA session per appointment (sessions ended following 1 min without responding, or after 1 hr).

As was the case during the PRA, researchers then began each session by saying, “3, 2, 1, start” and looking down to avoid eye contact, after paraphrasing the following contingency review.

*In this version of the game, you can earn (reinforcer) by emitting the target response (assigned schedule value) number of times like this (researcher models response requirement). You can work for as long as you want to earn (reinforcer); I've bought a lot of them and am happy to keep giving them to you. But it's important that you only respond for as long as you want (reinforcer). The moment you're full, tired of responding, bored, or just want to talk, you just need to say you're done by touching this card. I'll pay you no matter what, so only respond for as long as you want (reinforcer). Can you show me that you know how to say you're done?*

For participants who did not want to hear the entire contingency review, researchers simply asked them if they remembered what to do. If so, they asked participants to explain relevant contingencies to the researcher. If, during a session, participants asked how many responses were needed to earn the next reinforcer, the researcher told them. Likewise, researchers counted out loud the number of responses made by participants who needed this support for the first few reinforcer deliveries of a given session. Approximately once every 5 min, researchers also reminded participants that they could quit whenever they wanted to stop. Sessions terminated after 60 s elapsed without a target response, a participant stated that they wished to terminate the session, they touched a laminated “stop” card, they walked away from the table, or 1 hr had elapsed (no session was ever terminated due to the passage of time). The entire assessment ended after the aggregate response output for a current session fell below the output produced by the previous session (i.e., after *P*_*max*_ had been empirically confirmed).

### Economic measures and statistical methods

Target responding and reinforcers obtained in the Basis *x* PRA sessions were used to derive two economic measures: *BP1* (i.e., the across‐session average ratio value at which the final reinforcer was obtained) and *predicted P*_*max*_ (i.e., the across‐session average ratio value at which the highest response output occurred; e.g., Reed et al., 2009).

In the PFRA, measures derived from target responding and reinforcer delivery included *Q*_0_ (i.e., the number of freely available reinforcers consumed during the assessment of demand intensity), equilibrium at *FR 1* (i.e., reinforcers consumed during the FR 1 sessions of the PFRA), *observed O*_*max*_ (i.e., obtained peak response output), and *observed P*_*max*_ (i.e., the schedule value that yielded observed *O*_*max*_).

Importantly, we did not anticipate that small morsels of food or small units of sound would support large ratios of responding for this population. Thus, to avoid generating an unnecessarily narrow range of variability in consumption and responding within and across participants (Kaplan et al., 2018), specific unit prices in Basis *x* PRAs and PFRAs were intentionally low and step‐size progressions were arithmetic (as opposed to geometric).

Pearson correlation coefficients were used to explore the potential relations between obtained and derived measures across Basis *x* PRA and PFRA conditions. Preliminary diagnostic tests (scatterplots, *Q*‐*Q* plots, and residual plots) were used to assess linearity, residual variance, and the normality of residual distributions. Additionally, we conducted a studentized Breusch–Pagan test to evaluate the presence of heteroskedasticity (nonconstant residual variance). Due to significant heteroskedasticity, data point clusters in the scatterplots, and outlier data points (see Figures SM1 and SM2 in the Supporting Information), all dependent measures were natural log transformed prior to analysis. This remedied the noted violations and allowed us to use a consistent statistical method—Pearson correlation—for all variable pairs. Each correlation was tested for statistical significance using a *t* test. As the study involved planned comparisons between 5 Basis *x* PRA and 12 PFRA variables—resulting in a total of 60 statistical tests, the risk of Type I error inflation was a concern. To adjust for multiple testing, we applied the false discovery rate (FDR) correction (Benjamini & Hochberg, 1995). This procedure ranks the obtained *p* values and adjusts each one based on its rank and the total number of tests, ensuring a balance between sensitivity (detecting true effects) and specificity (controlling for false positives).

## RESULTS

Demand curves for individual participants derived from Basis *x* PRA and PFRA are displayed in Supporting Information Figures SM3–8. Because assessments of demand intensity call for unconstrained access to programmed reinforcers, consumption at *Q*_0_ was plotted on PFRA graphs but not Basis *x* PRA graphs.

In a deviation from conventional graphing standards, we did not plot our demand‐curve analyses on double‐logarithmic coordinates in Supporting Information Figures SM3–8 and instead used individual participant performances to establish relevant scales. Specifically, we used the highest *y*‐axis value produced by either assessment to set the ceiling for all *y*‐axes of a given participant. Likewise, the highest *x*‐axis value produced by either assessment was used to set the range for all *x*‐axis scales of a given participant. We did this to keep our analysis focused on participant performances at market prices (Kaplan et al., 2018), in this case, to help readers attribute meaning to white spaces in graphs (i.e., because scales were individualized but held constant across graphs for each participant, taller data paths can be interpreted as higher equilibrium points and wider data paths can be interpreted as greater inelasticity).

Relevant descriptive statistics are summarized in Table 1. The mean *BP1* produced by Basis *x* PRA was 16.31 (range: 1–56), and the mean *P*_*max*_ predicted by Basis *x* PRA was 14.63 (range: 1–61). The mean *P*_*max*_ directly observed during the PFRA comparison was FR 8.19 (range: 1–34).

**TABLE 1:** Descriptive statistics for dependent variables obtained from Basis × PRA and PFRA.

	Dependent variables	Mean	*SD*	Median	Range
**Basis *x* PRA**					
Session statistics	Mean R per Session	153.3	217.5	57.5	1–1,096.67
	Mean S^R^ per Session	11.34	8.22	8.67	1–38
	Mean Session Duration (s)	530.55	567.25	336.67	19.33–3,235.33
Assessment statistics	Total R	463.41	656.36	172.5	3–3,290
	Total S^R^	34.22	24.88	26	3–114
	Total Duration (s)	1,792.25	1,810.9	1,165.5	184–10,456
Economic metrics	Mean *BP1* (Observed)	15.7	12.31	11	1–56
	*P*_*max*_ (Observed)	14.59	10.97	13	1–61
**PFRA**					
*Q*_0_ statistics	Mean S^R^ per Session	37.27	50.57	17.5	1–342
	Mean Session Duration (s)	374.56	534.74	210.5	13–3,600
FR 1 statistics	Mean R per Session	35.29	39.93	26.5	1–215
	Mean S^R^ per Session	35.29	39.98	26.5	1–215
*P*_*max*_ statistics	*Pmax* (Observed)	8.44	5.02	7	1–34
	Mean R per Session	217.83	284.81	122.5	2–1,551
	Mean S^R^ per Session	29.98	38.14	16	1–251
	Mean Session Duration (s)	697.51	744.73	416.5	29–3,600
Assessment statistics	Total Sessions	5.61	1.8	5	3–14
	Total R	618.8	851.41	336	3–5,692
	Total S^R^	250.73	1,397.91	75	2–13,752
	Total Duration (s)	2,731.38	3,065.94	1,817.5	76–15,285

### Correlations

Table 2 reports Pearson correlation coefficients and FDR‐adjusted *p* values used to evaluate the relation between Basis *x* PRA and PFRA outcomes. Across the top of Table 2, columns correspond to dependent measures averaged across all Basis *x* PFRA test sessions. The economic metrics columns refer to *BP1* and *P*_*max*_ values derived for individual subjects' across‐sessions Basis *x* PRA data set. For comparison, the farthest right column also includes data from PFRA‐observed *P*_*max*_. Although this final variable was obtained from PFRA and not Basis *x* PRA, we juxtaposed it with economic metrics derived from Basis *x* PRA *P*_*max*_ to facilitate the comparison for readers interested in determining which metric might be most predictive of demand equilibrium across the largest range of circumstances.

**TABLE 2:** Pearson correlation coefficients and corresponding *p* values.

		Full Aanalysis (Basis *x* PRA)			Economic metrics		
		Total R	Total S^R^	Total duration (s)	PRA‐BP1	PRA‐predicted *P*_*max*_	PFRA‐observed *P*_*max*_
**NCR (PFRA)**	Mean S^R^	*r = *.40 *p = *.00***	*r = *.40 *p = *.00***	*r = *.39 *p = *.00***	*r = *.38 *p = *.00***	*r = *.33 *p = *.00***	*r =* −.09 *p =* .52
	Mean duration (s)	*r = *.30 *p = *.00***	*r = *.29 *p = *.01*	*r = *.45 *p = *.00***	*r = *.27 *p = *.01*	*r = *.24 *p = *.02*	*r =* −.17 *p =* .17
**FR 1 (PFRA)**	Mean R	*r = *.82 *p = *.00***	*r = *.82 *p = *.00***	*r = *.77 *p = *.00***	*r = *.82 *p = *.00***	*r = *.79 *p = *.00***	*r =* −.05 *p =* .69
	Mean S^R^	*r = *.83 *p = *.00***	*r = *.83 *p = *.00***	*r = *.77 *p = *.00***	*r = *.82 *p = *.00***	*r = *.79 *p = *.00***	*r =* −.05 *p =* .69
**Observed *P***_***max***_ **(PFRA)**	P_max_ (observed)	*r = *.07 *p = *.53	*r = *.08 *p = *.52	*r =* −0.08 *p = *.52	*r = *.09 *p = *.46	*r = *.07 *p = *.54	N/A
	Mean R	*r = *.76 *p = *.00***	*r = *.75 *p = *.00***	*r = *.61 *p = *.00***	*r = *.75 *p = *.00***	*r = *.74 *p = *.00***	*r =* .41 *p =* .00***
	Mean S^R^	*r = *.78 *p = *.00***	*r = *.77 *p = *.00***	*r = *.71 *p = *.00***	*r = *.76*** *p = *.00	*r = *.76 *p = *.00***	*r =* −.19 *p =* .13
	Mean duration (s)	*r = *.64 *p = *.00***	*r = *.63 *p = *.00***	*r = *.79 *p = *.00***	*r = *.63 *p = *.00***	*r = *.64 *p = *.00***	*r =* −.03 *p =* .77
**Full analysis (PFRA)**	Total sessions	*r =* −0.01 *p = *.92	*r =* −0.01 *p = *.95	*r =* −0.08 *p = *.52	*r = *.01 *p = *.94	*r =* −0.04 *p = *.76	*r =* .86 *p =* .00***
	Total R	*r = *.72 *p = *.00***	*r = *.72 *p = *.00***	*r = *.57 *p = *.00***	*r = *.72 *p = *.00***	*r = *.69 *p = *.00***	*r =* .55 *p =* .00***
	Total S^R^	*r = *.80 *p = *.00***	*r = *.80 *p = *.00***	*r = *.72 *p = *.00***	*r = *.79 *p = *.00***	*r = *.78 *p = *.00***	*r =* .23 *p =* .07
	Total duration (s)	*r = *.61 *p = *.00***	*r = *.60 *p = *.00***	*r = *.74 *p = *.00***	*r = *.60 *p = *.00***	*r = *.58 *p = *.00***	*r =* .25 *p =* .03*
	Average	*r =* .56	*r =* .56	*r =* .53	*r =* .55	*r =* .53	*r =* .16

*Note*: Highlighted cells reflect correlations that were not statistically significant.

* *p* < .05;

*** *p* < .001.

The left‐most columns correspond to obtained and derived outcomes obtained in individual PFRA sessions. The bottom rows of those left‐most columns correspond to across‐participant averages. Statistically significant correlations are marked with asterisks, and shaded cells highlight correlations that were not statistically significant. Average correlation coefficients at the bottom of each column are intended to summarize the adequacy of each Basis *x* PRA measure for predicting all listed PFRA outcomes.

**FIGURE 2:**
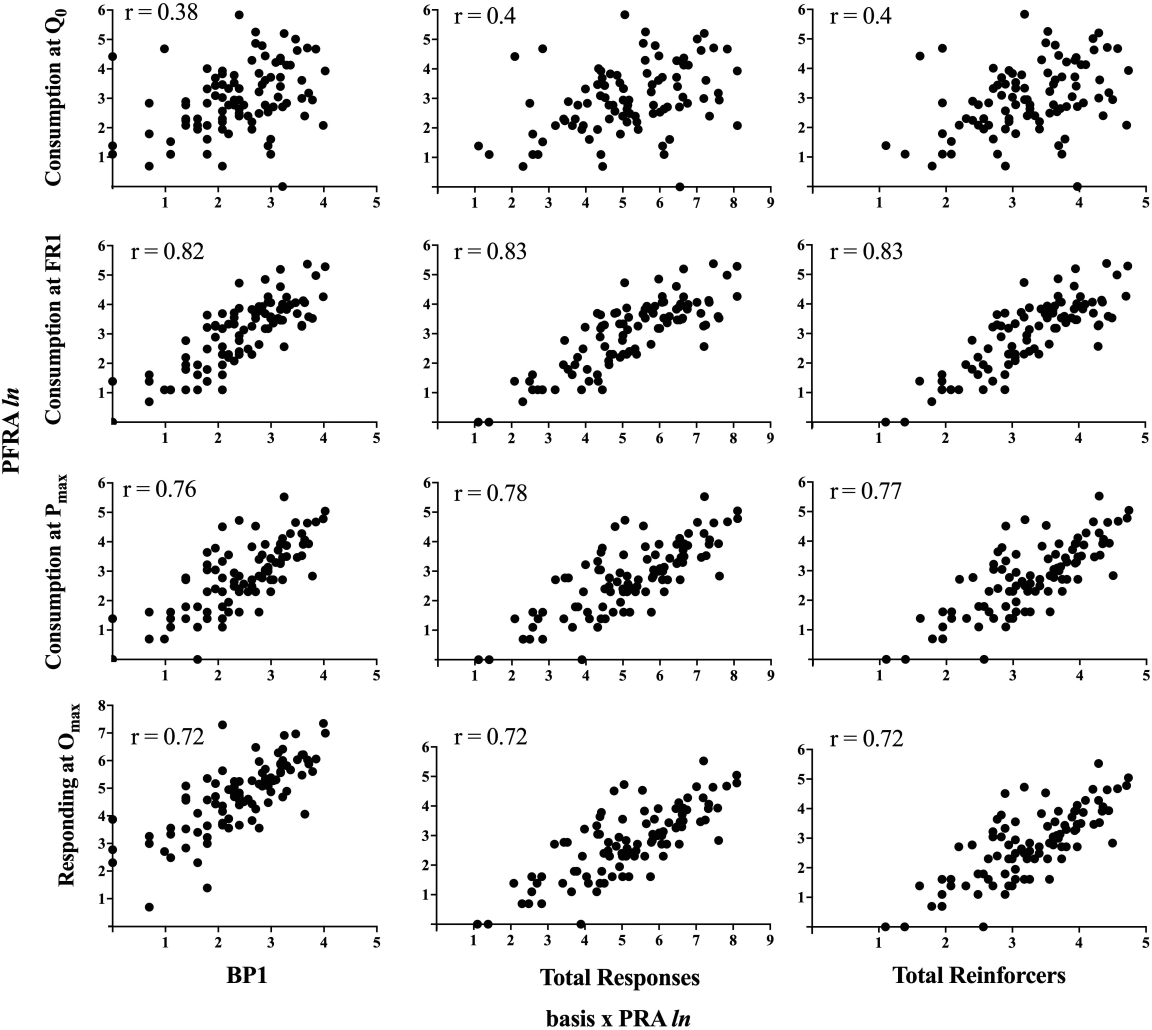
Log‐transformed correlations between Basis *x* PRA‐*BP1* (left column), Basis *x* PRA‐total responses emitted (middle column), Basis *x* PRA‐total reinforcers consumed (right column) and PFRA‐consumption at *Q*_0_ (top row), PFRA‐consumption at FR 1 (second row), PFRA‐consumption at *P*_*max*_ (third row), and PFRA‐total responding at *O*_*max*_ (bottom row). PRA = progressive‐ratio analysis; PFRA = progressive‐fixed‐ratio analysis, *ln* refers to the natural log conversion of the dependent measures.

Figure 2 shows scatterplots of several of the Table 2 relations of interest between Basis *x* PRA and PFRA dependent measures. Because the three Basis *x* PRA measures were strongly correlated, the Pearson *r* values across each row are very similar. These Basis *x* PRA measures were significantly positively correlated with the two measures of demand intensity: consumption at *Q*_0_ and at FR 1. Likewise, they were significant predictors of PFRA reinforcer consumption at *P*_*max*_ and responding at *O*_*max*_.

**FIGURE 3:**
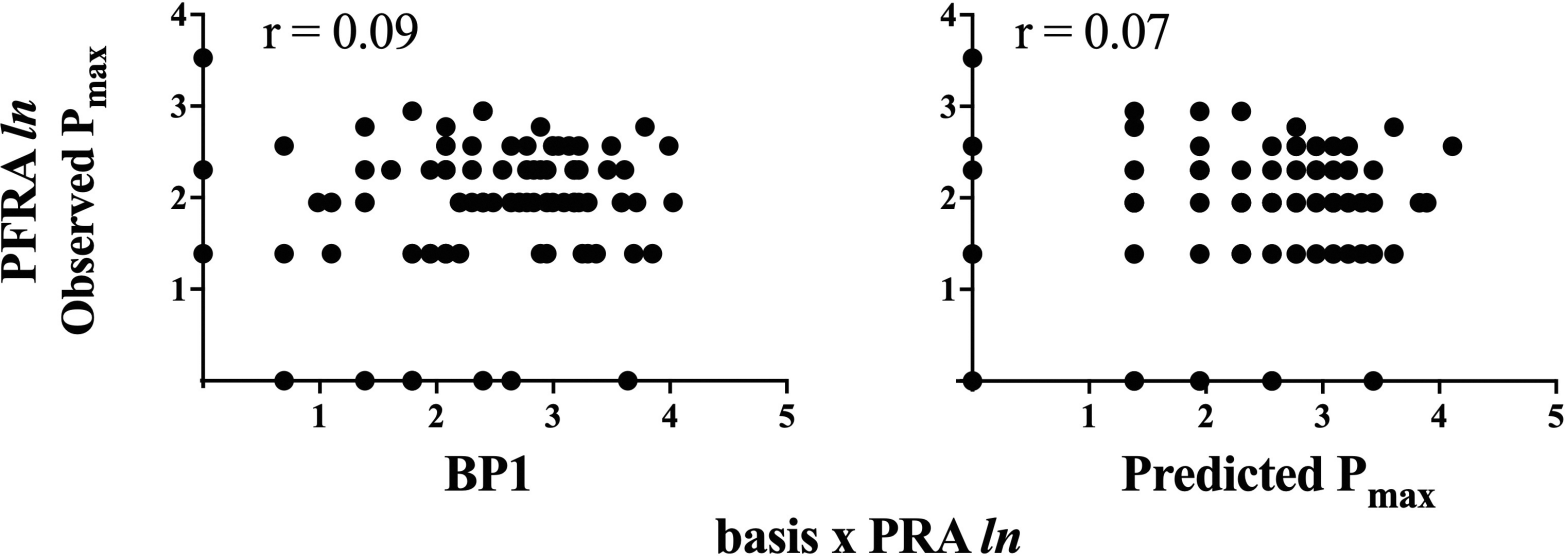
Log‐transformed correlations between PFRA‐observed P_max_ (*y*‐axis) and Basis *x* PRA‐observed *BP1* and Basis *x* PRA‐observed *P*_*max*_ (*x*‐axes in left and right panels, respectively; *ln* refers to the natural log conversion of the dependent measures).

Figure 3 shows correlations between measures of demand elasticity (*BP1* and predicted *P*_*max*_) derived from Basis *x* PRA and *P*_*max*_ values obtained in the PFRA sessions. Neither *BP1* nor *P*_*max*_ values derived from Basis *x* PRA sessions were significantly correlated with obtained *P*_*max*_ values from PFRA sessions.

**FIGURE 4:**
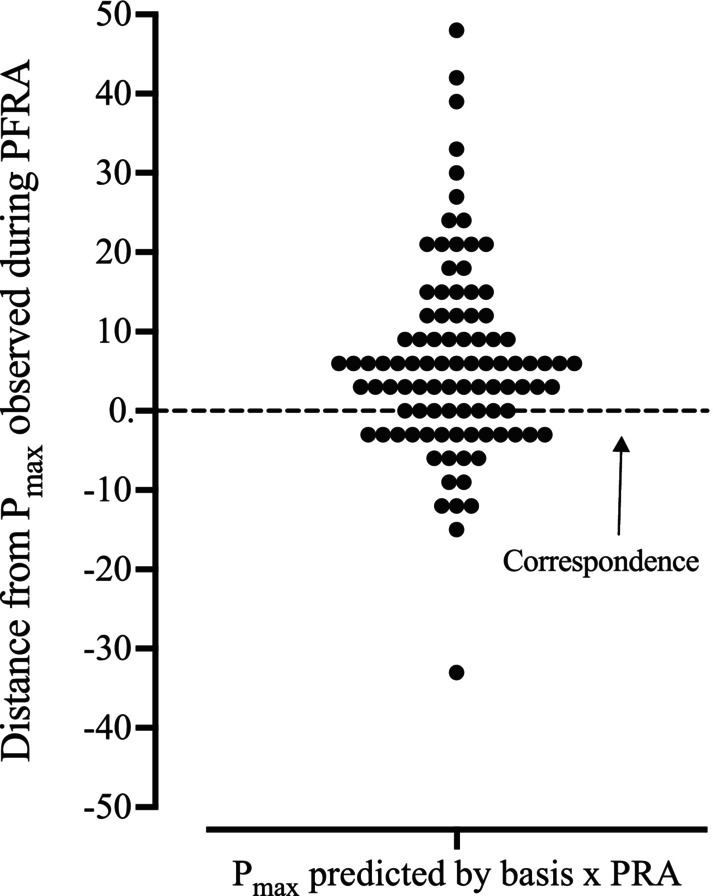
Distance of *P*_*max*_ predicted by Basis *x* PRA from *P*_*max*_ established by PFRA.

Specifically, and as displayed in Figure 4, Basis *x* PRA accurately predicted *P*_*max*_ across 8.3% (8 of 96) of cases. Basis *x* PRA overestimated *P*_*max*_ for 66.7% (64 of 96) of cases and underestimated *P*_*max*_ for 25% (24 of 96) of cases. The modal difference in unit price between Basis *x* PRA predictions of *P*_*max*_ and *P*_*max*_ observed during PFRA was 6 (2 steps).

## DISCUSSION

In reference to observational measurement systems, validity refers to the degree to which the uses and purposes of data outputs are supported by evidence and theory (Yoder et al., 2018). Although there are various forms of validity (e.g., content, construct, social), criterion‐related validity refers to the degree to which a given empirical output that is theoretically related to a variable of interest corresponds to a more established, and ideally direct, measure of that variable (Yoder et al., 2018).

For the purposes of demand‐curve analysis, data obtained from PFRA and Basis *x* PFRA offer a direct measure of the equilibrium between supply and demand because reinforcers are available indefinitely and sessions only terminate after participants choose to stop responding. In contrast, the validity of deriving economic metrics from PRA or Basis *x* PRA is neither automatically nor logically entailed because within‐session response‐dependent increases in ratio requirements introduce a second, potentially suppressive, contingency that artificially caps the amount of responding and reinforcer consumption possible at each unit price (thus imposing an artificial ceiling). Nevertheless, the practical advantages of PRA and Basis *x* PRA over PFRA render them attractive substitutes for demand‐curve analysis in applied research paradigms—provided their contribution to demand quantification can be clearly established. In the service of this goal, the purpose of this study was to evaluate whether economic metrics of elasticity (i.e., *P*_*max*_) and equilibrium (i.e., demand intensity, *O*_*max*_) derived from Basis *x* PRA outcomes were predictive of those observed during PFRA demand‐curve analysis.

Our findings were multifaceted. First, neither *BP1* nor predictions of *P*_*max*_ obtained from Basis *x* PRA corresponded with *P*_*max*_ values observed during PFRA. Further, *BP1* was *lower than* the observed *P*_*max*_ for 28.1% of cases (27 of 96). Consequently, our data suggest that it is likely invalid to use Basis *x* PRA to make predictions about how specific unit prices will influence patterns of responding and consumption (e.g., *P*_*max*_, *BP1*). That is, Basis *x* PRA reliably predicted neither unit prices capable of optimizing response output (i.e., *P*_*max*_) nor the unit prices beyond which programmed consequences stop functioning as reinforcers (i.e., *BP1*).

**FIGURE 5:**
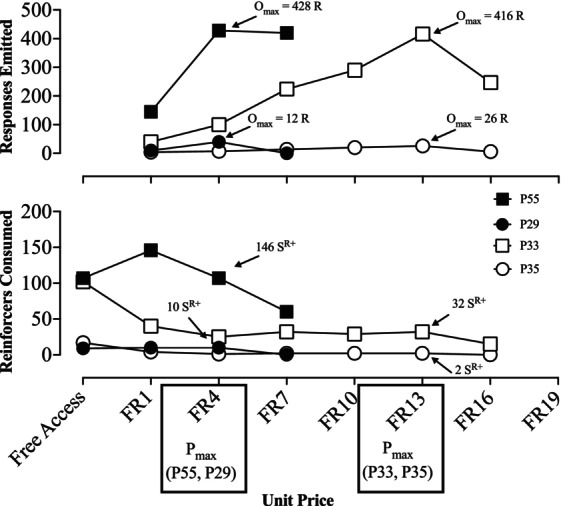
Representative performance patterns from PFRA assessments. R = responses; S^R+^ = reinforcers.

A second, and intriguing, finding of our study was that Basis *x* PRA outputs were significantly correlated with equilibrium points from the PFRA (i.e., demand intensity, response and reinforcer outputs during FR 1 and *P*_*max*_). In fact, *BP1* from Basis *x* PRA was a better predictor of equilibrium across the unit prices sampled during PFRA than PFRA‐observed *P*_*max*_ (Table 2). To exemplify this point, Figure 5 displays the PFRA results of four participants with representative but distinctive performance patterns. Specifically, both P29 and P55 had low PFRA‐observed *P*_*max*_ values (i.e., FR 4) and P33 and P35 both had high PFRA‐observed *P*_*max*_ values (i.e., FR 13). Despite *P*_*max*_ parity across each of these two sets of participant dyads, it is instructional to see that *O*_*max*_ values were most similar for participants with different *P*_*max*_ values. Specifically, both P55 and P33 had *O*_*max*_ values that exceeded 400 responses. Likewise, both P29 and P35 had *O*_*max*_ values lower than 30 responses. For P35 (high *P*_*max*_ value), demand at *P*_*max*_ *never* exceeded demand from P55 (low *P*_*max*_ value).

What we learn from Figure 5 is that high‐equilibrium reinforcers (i.e., those with high demand intensities or that support high *O*_*max*_ values) can sometimes be optimally priced at lower response requirements. Likewise, low‐equilibrium reinforcers (i.e., those that support low demand intensities or low *O*_*max*_ values) can sometimes be optimally priced at higher response requirements. Although it was sometimes the case in our study that a low *P*_*max*_ corresponded with low equilibrium points and a high *P*_*max*_ corresponded with high equilibrium points (e.g., P33 and P29), the fact that performance profiles could also mirror those typified by P55 and P35 demonstrates that the variables that influence equilibrium are different from those that determine elasticity. This fact should discourage the practice of using *P*_*max*_ as an index of reinforcer efficacy (e.g., Gilroy et al., 2021; Reed et al., 2009). Said more plainly, our results suggest *P*_*max*_ does not effectively predict how much behavior a given reinforcer will support; it just identifies the schedule parameters at which peak response output is obtained. In contrast, Basis *x* PRA‐*BP1* does not identify the schedule parameters at which peak response output is obtained; rather, it predicts how much behavior a given reinforcer is likely to support.

Relevant to this point, some researchers have suggested that measures of equilibrium, like demand intensity and *O*_*max*_, offer the most useful measure of reinforcer efficacy (e.g., Hursh & Winger, 1995) and have demonstrated that nonnormalized *O*_*max*_ values will often correspond with PR breakpoints (e.g., Johnson & Bickel, 2006; Madden et al., 2007a, 2007b; Rodefer & Carroll, 1997). Our own results appear to extend these findings by demonstrating that Basis *x* PRA‐*BP1* correlates with not only *O*_*max*_ but also response and reinforcer outputs at unit prices other than *P*_*max*_ (i.e., NCR, FR 1)—suggesting the results of Basis *x* PRA are likely to be relevant across a range of circumstances (not just those precisely situated within demand‐curve analysis). That is, Basis *x* PRA‐*BP1* did not implicate optimal pricing conditions but did consistently predict high and low equilibrium points across circumstances (i.e., NCR, FR 1, *P*_*max*_). This fact could have applied implications.

For example, Lambert et al. (2024) found that reinforcer consumption (relative to equilibrium) was the variable that most consistently exacerbated, and mitigated, extinction bursts. Specifically, individuals whose consumption prior to extinction had not approximated equilibrium were more likely to both persist (as evidenced by differentially high response and latency outputs) and escalate (as evidenced by proportion‐of‐baseline measures and the prevalence of bursting) prior to response elimination.

Similarly, substance‐use research has reliably demonstrated that demand intensity and *P*_*max*_ can be predictive of treatment success for certain intervention programs. That is, participants who consumed larger quantities of alcohol (Murphy et al., 2015) and nicotine (Mackillop et al., 2016) at *Q*_0_ and/or who worked harder to defend *Q*_0_ consumption patterns in the face of rising costs were also more likely to relapse when competing incentives were arranged to disrupt consumption.

Given these findings, it may be that functional reinforcers for challenging behavior with high equilibrium points will be more persistent in the face of extinction and more susceptible to treatment relapse than functional reinforcers that yield low equilibrium points. It may thus be that the extinction of responding maintained by “high equilibrium” reinforcers is more likely to be contraindicated. Consequently, future research should explore the extent to which value outputs that correlate with equilibrium levels (e.g., *BP1*) that can be derived from efficient assessments (e.g., PRA or Basis *x* PRA) and can easily be imbedded into standard‐of‐care procedures (e.g., functional analysis) are predictive of treatment success (cf. Hagopian et al., 2018).

A few of this study's limitations should be noted. First, our analyses did not control for commodity type (i.e., some participants earned edibles, others earned music or crafts). Second, although we attempted to conduct all analyses in a closed economy, we could not guarantee that this occurred. Variability in either or both procedures is likely to have differentially influenced outcomes across participants. However, because both economy type and reinforcer type were held constant for each participant, within‐subject comparisons remained unaffected. That is, within each participant, Basis *x* PRA and PFRA results would have been equally influenced by idiosyncratic characteristics of the programmed reinforcer in either a closed or an open economy. Thus, because our primary dependent variable was within‐subject correspondence of economic metrics produced by Basis *x* PRA and PFRA, our findings appear to retain validity despite the potential for uncontrolled variability between subjects.

Third, for practical reasons, we terminated PFRAs after *P*_*max*_ was identified and never established either *BP1* or *BP0*. Consequently, our PFRA preparation more thoroughly assessed the influence of unit prices that contributed to inelastic demand than that of unit prices that contributed to elastic demand (there was a similar limitation for our Basis *x* PFRA preparation). Fourth, Basis *x* PRA was always conducted prior to PFRA. Thus, it is possible that order effects skewed our results. Specifically, participants may have been more susceptible to burnout and/or boredom during PFRA because the experimental arrangement ensured that this assessment always came second. Fifth, because we compensated participants for their time, it is possible that responding was sensitive to broader monetary contingencies (i.e., participants could have earned more money by extending time in sessions). Consequently, future researchers might consider alternative methods of compensation that are fair but disentangled from session performance.

Sixth, we did not establish steady‐state responding for each unit price in our PFRA demand‐curve analysis. Importantly, Basis *x* PFRA is prohibitively time consuming (Leon et al., 2021) and would have precluded our ability to conduct this study. Because there is established empirical and conceptual precedent for rapid assays that leverage PFRA (e.g., Madden et al., 2005; Raslear et al., 1988), we opted to employ this abbreviated methodology. Thus, all conclusions drawn from this study should be considered tentative. Given that there would be substantial practical and ethical challenges to conducting Basis *x* PFRAs with humans, future research could systematically replicate this study using more rigorous methods targeting nonhuman populations.

As a final note, it was peculiar that reinforcer consumption increased (instead of decreased) when transitioning from NCR to FR 1 during PFRA for 46.9% (45 of 96) of participants. Although it is common to exclude such data prior to conducting a demand‐curve analysis, we opted to keep it for two reasons. First, this pattern was not restricted to outliers but rather was observed for a substantial minority of participants. Given the phenomenon's prevalence, we could not justify omission. Second, there is evidence in the applied literature to suggest that the introduction of contingencies, in and of itself, possesses reinforcing properties (e.g., Luczynski & Hanley, 2009, 2010). That reinforcer consumption increased following the introduction of low‐effort contingencies for so many participants is potentially instructional and could have implications for future research efforts.

Despite the limitations, this study is unique in its scope and scale and offers future research important data that specifies whether and the ways in which PRAs might reasonably be used to quantify demand in applied populations. To summarize, our study suggests that PRAs can predict some but perhaps not all economic metrics typically obtained from demand‐curve analysis. Specifically, researchers are most likely to find success when interpreting Basis *x* PRA breakpoints as an index of a reinforcer's demand intensity rather than as an index of optimal pricing conditions.

## AUTHOR CONTRIBUTIONS

Joseph M. Lambert led project conceptualization, implementation, data management, data analysis, and writing. Maria A. Osina assisted with project conceptualization, data management, and writing. Johanna L. Staubitz assisted with project conceptualization and writing. Derek D. Reed assisted with project conceptualization and writing. Gregory J. Madden assisted with project conceptualization and writing.

## CONFLICT OF INTEREST STATEMENT

All authors declare no conflicts of interest.

## ETHICS APPROVAL

This study was preregistered on clinicaltrials.gov (NCT04842500), adhered to ethical guidelines, and was approved by Vanderbilt's IRB (IRB #210691). Informed consent was obtained from all participants and data have been anonymized to protect participant privacy and confidentiality.

## Supporting information


**Data S1** Supporting Information

**Data S2** Supporting Information

**Data S3** Supporting Information

**Data S4** Supporting Information

**Data S5** Supplementary Data

## Data Availability

The data that support the findings of this study are available from the corresponding author upon reasonable request.
