# Supplementary material for: Evaluating contributions of progressive ratio analysis to economic metrics of demand
Source: J Exp Anal Behav. 2025 Dec 26;125(1):e70077. doi: 10.1002/jeab.70077 (PMC12742636; doi:10.1002/jeab.70077)
Supplement: Supplementary file 1 — Data S1 Supporting Information [file JEAB-125-0-s002.docx]

**Appointment Mechanics and Procedural Fidelity Sheets**

Appointment Mechanics

**Prior to the appointment**

1. Prepare reinforcers (e.g., equate size/quantity) before going to appointment and ensure you have a sufficient amount
2. Call to let relevant stakeholders know that you are coming and confirm they can receive you
3. Drive to location
4. Say “hi” to everyone (smile and respond to comments in contextually appropriate ways)
5. Set up your materials
6. Invite relevant participant to come work with you
7. Initiate a 5-min casual conversation with participant before you begin any assessment

**During the appointment**

1. Display apparatus for the target response, a low-preferred alternative activity, and a “stop” card.
2. Paraphrase the following statement:

“We are going to start a session, now. This just means I’m going to give you an opportunity to earn (reinforcer). During sessions I cannot talk to you. That’s just a rule I have to follow. It doesn’t mean I don’t want to talk or that I’m not happy. If you want (reinforcer) after the session starts, you can work on (target response). If you don’t want (reinforcer), or would rather talk to me, you only need to stop (target response) for one minute. You can also just say, ‘I’m done’ or point to this stop card. While you wait, you can (alternative activity) if you like.”

1. Initiate relevant assessment. Document outcomes as needed.
2. When the appointment is over, calculate the number of “punches” the participant has earned, administer them, and have both you and participant initial relevant cells in payment tracker.
3. (When relevant) pay participant gift cards.

**After the appointment**

1. Clean up your area
2. Let everyone know you’re leaving and say “good bye”
3. Go somewhere with the internet (coffee shop, campus, your house).
4. Calculate IOA and PF (when relevant)
5. Update paper data summary files in participant binder (if this hasn’t already been done), update EDS on Box, transfer raw Countee data files to relevant Box folders.
6. Attend to “reserves” (e.g., number of reinforcers remaining) and document need for purchases when need is anticipated.

Debriefing Statement Following Study Completion

“That last session was a little weird, wasn’t it? Did you notice that I stopped giving you (reinforcer) when you (response)? The truth is, that wasn’t an accident. This entire study was to help me understand how much work you would do before you stopped when I stopped (reinforcer). You did great and I learned so much! Now that the study is over, you won’t have to (response) anymore, isn’t that great! Since this was the last time you’ll be working on this project, let’s take a look at you punch card and see how much money I still owe you.”

**Participant Compensation Tracking Sheet**

Participant ID: ____________________

Gift Card Preference

Amazon Walmart Other: __________

| Date | Assent  (“Do you want to work with me today?”) | # of Holes Punched During Appt.  (20 min = 1 punch) | Punch Card Completed (i.e., 10 punches)  Y/N | $25 Gift Card Paid after 10 punches  Y/N | Experimenter Initial | Participant Initial |
| --- | --- | --- | --- | --- | --- | --- |
|  |  |  |  |  |  |  |
|  |  |  |  |  |  |  |
|  |  |  |  |  |  |  |
|  |  |  |  |  |  |  |
|  |  |  |  |  |  |  |
|  |  |  |  |  |  |  |
|  |  |  |  |  |  |  |
|  |  |  |  |  |  |  |
|  |  |  |  |  |  |  |
|  |  |  |  |  |  |  |
|  |  |  |  |  |  |  |
|  |  |  |  |  |  |  |
|  |  |  |  |  |  |  |
|  |  |  |  |  |  |  |
|  |  |  |  |  |  |  |
|  |  |  |  |  |  |  |
|  |  |  |  |  |  |  |
|  |  |  |  |  |  |  |
|  |  |  |  |  |  |  |
|  |  |  |  |  |  |  |
|  |  |  |  |  |  |  |


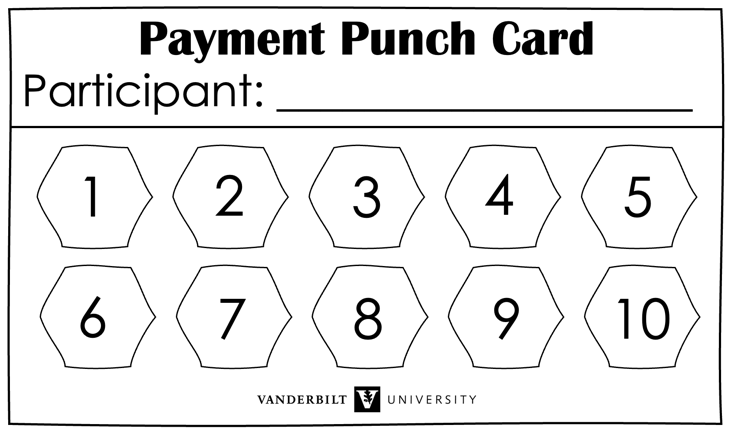

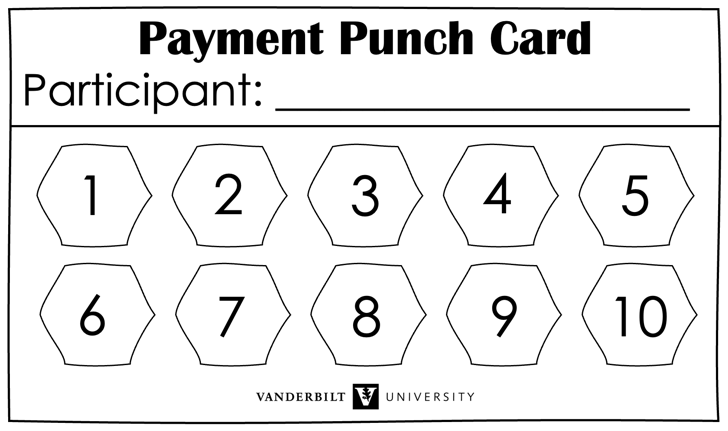


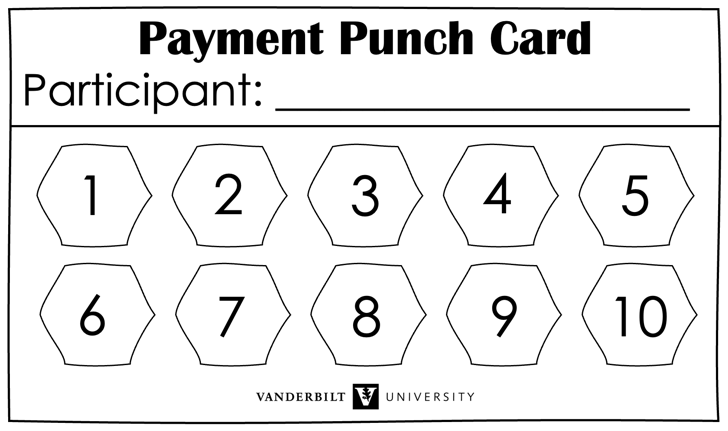

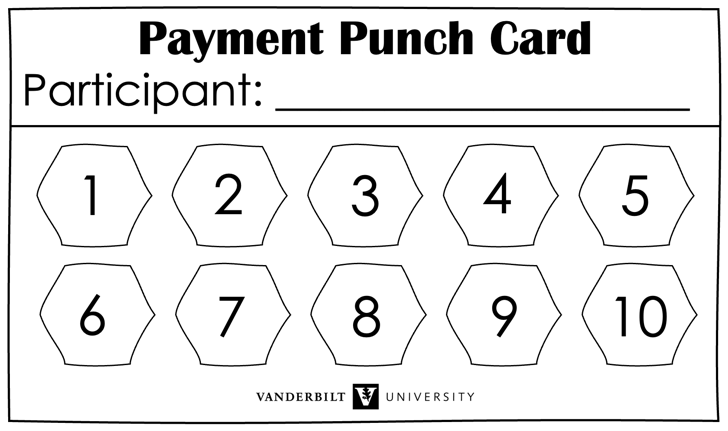


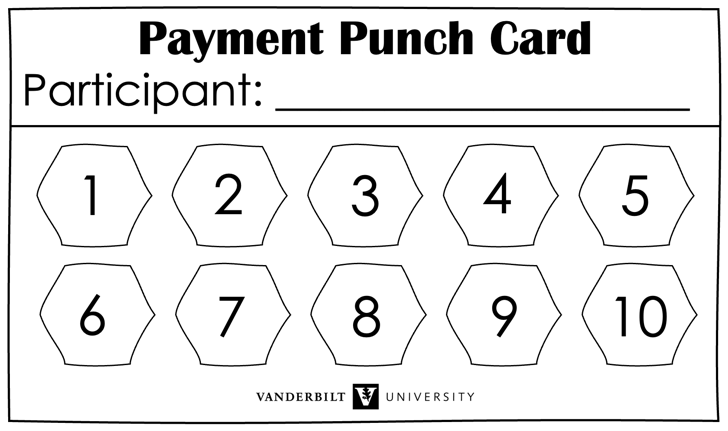

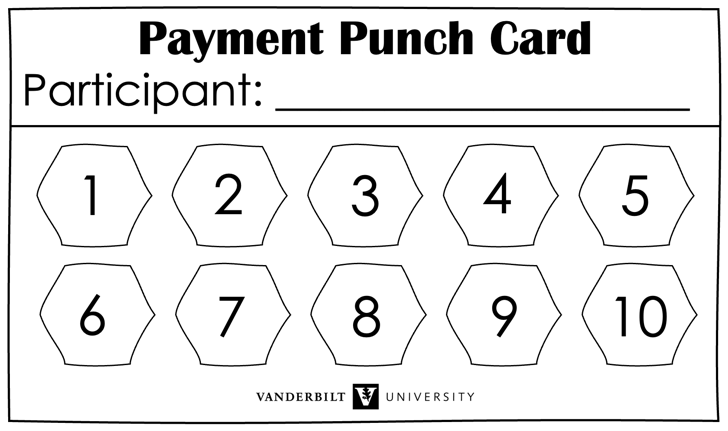


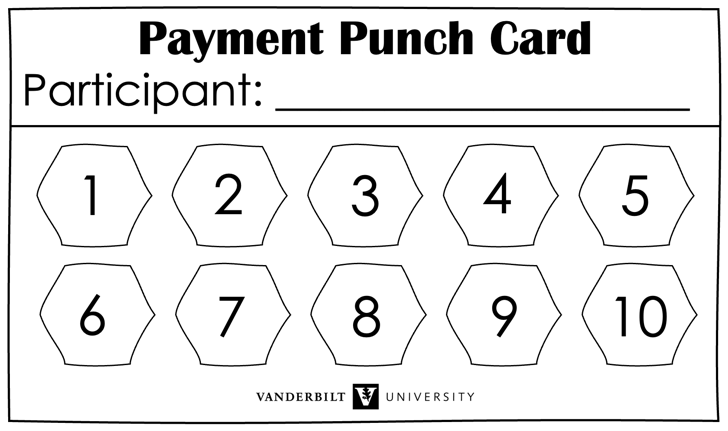

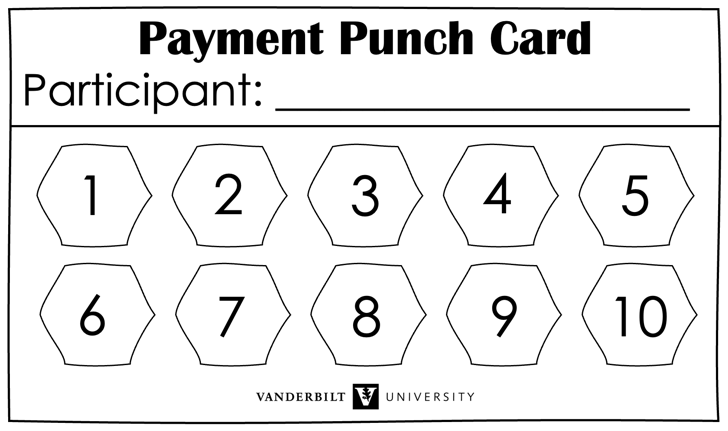


**Procedural Fidelity (Free Access to Target Reinforcer)**

Date: Session:

Participant #: Observer initials (prim/reli):

| **Procedure** | **Yes** | **No** | **N/A** |
| --- | --- | --- | --- |
| 1. Extraneous materials were removed from assessment area |  |  |  |
| 1. Session termination card and alternative stimulus present. |  |  |  |
| 1. Facilitator paraphrased (a) *“Which of these would you most like to work for?*” (b) presented an array of edible options and (c) allowed to select their highest preferred item. |  |  |  |
| 1. After edible selection, facilitator paraphrased the following statement: *“Wonderful. We’ll start working for this the next time I come. Right now, I’m just going to let you have as much of it as you want. Each time you take one (point) and eat it, I’ll replace it with another. When you don’t want to eat anymore don’t take anymore. Remember, you can always choose to play with this (low-preferred alternative) or tell me that you’re done or that you’d rather talk to me than eat these.”* |  |  |  |
| 1. A plate was available for placing a single morsel of selected edible item. |  |  |  |
| 1. A single morsel rested on the plate at all times. Each time a morsel was consumed, it was replaced. |  |  |  |
| 1. All morsels consumed were approximately the same size, shape, and quality |  |  |  |
| 1. The edibles “reserve” (i.e., the container from which edibles were replaced) was controlled by the implementer and concealed to the extent to which concealment was possible. |  |  |  |
| 1. The session terminated either after 1 min of no consumption, or after the participant indicated they wanted the session to end. |  |  |  |
| Total |  |  |  |
| Yes / (Yes + No) = |  | | |

**Procedural Fidelity (Target Response Training)**

Date: Session:

Participant #: Observer initials (prim/reli):

Corrective prompt (circle one):

Manual guidance Model Gesture Other

| **Procedure** | **Yes** | **No** | **N/A** |
| --- | --- | --- | --- |
| 1. Extraneous materials were removed from assessment area |  |  |  |
| 1. Session termination card and alternative stimulus present. |  |  |  |
| 1. A plate upon which edible morsels of a **non-target reinforcer** could be placed was available. |  |  |  |
| 1. Facilitator presented only one task-candidate at a time. |  |  |  |
| 1. Facilitator paraphrased, *“Show me if you can do this.”* Facilitator then modeled an action for the participant to imitate which entailed both a manipulation (e.g., open the wallet) and a restoration (e.g., close the wallet), and then waited for the participant to attempt it. |  |  |  |
| 1. Facilitator discarded difficult tasks and continued to assess easily completed tasks. |  |  |  |
| 1. For easily completed tasks, facilitator paraphrased the following statement: *“You can earn (****non-target reinforcer)*** *by doing (task). Try it”* Upon task completion, facilitator delivers morsel of (**non-target reinforcer).** *Facilitator can modify instructional procedure as needed. Please document all modifications.* |  |  |  |
| 1. Facilitator paraphrased the following statement: *“Okay, let’s practice. Every time you want more of (****non-target reinforcer),*** *do (task) one time. I will only give you one (****non-target edible reinforcer)*** *at a time, so you have to eat the ones I give you before you’ll get another one. Let’s try.”* After instruction, present a trial during with no prompting. Upon each task completion, facilitator delivered morsel of (**non-target edible reinforcer).** |  |  |  |
| 1. Facilitator discarded both tasks and reinforcers for which independent responses did not occur. |  |  |  |
| 1. Facilitator operationally defined as the target task (below) an action for which independent responses occurred. |  |  |  |
| Total |  |  |  |
| Yes / (Yes + No) = |  | | |

**Operational Definition of Target Task:**

**Procedural Fidelity (PRA Control)**

Date: Session #:

Participant #: Observer initials (prim/reli):

| **Procedure** | **Yes** | **No** | **N/A** |
| --- | --- | --- | --- |
| 1. Extraneous materials were removed from assessment area. |  |  |  |
| 1. Session termination card and alternative stimulus present. |  |  |  |
| 1. A plate upon which edible morsels of the **target reinforcer** could be placed was available. |  |  |  |
| 1. Facilitator paraphrased the following statement:   *“We are going to start a session, now. This just means I’m going to give you an opportunity to (task). When you* *do this (model task for participant), nothing is going to happen, so only do it if you like doing it.*  *If it’s boring, don’t do it. During sessions I cannot talk to you. That’s just a rule I have to follow. It doesn’t mean I don’t want to talk or that I’m not happy. If you don’t want to wait, or would rather talk to me, you only need to stop (target response) for one minute. You can also just say, ‘I’m done’ or point to this stop card. While you wait, you can (alternative activity) if you like.”* |  |  |  |
| 1. Each time participant responds the facilitator paraphrases the following and then restarts the session   *Remember, right now I just need to know that you know that responding will not get you anything. The way that I’ll know that is when you stop responding for one minute. While you wait you can play with (low-preferred alternative). Or, if you’re bored and don’t want to wait anymore, you can say “all done” and we’ll just end the sessions.* |  |  |  |
| 1. Control condition continues until either participant does not respond for 1 consecutive minute, or after the participant indicated they wanted the session to end. |  |  |  |
| Total |  |  |  |
| Yes / (Yes + No) = |  | | |

**Procedural Fidelity (PRA Test)**

Date: Session #:

Participant #: Observer initials (prim/reli):

| **Procedure** | **Yes** | **No** | **N/A** |
| --- | --- | --- | --- |
| 1. Extraneous materials were removed from assessment area |  |  |  |
| 1. Session termination card and alternative stimulus present. |  |  |  |
| 1. A plate upon which edible morsels of the **target reinforcer** could be placed was available. |  |  |  |
| 1. Facilitator paraphrased the following statement:   *“We are going to start a session, now. This just means I’m going to give you an opportunity to earn (reinforcer). At first it will be easy to earn (reinforcer), then it will take more time.*  *During sessions I cannot talk to you. That’s just a rule I have to follow. It doesn’t mean I don’t want to talk or that I’m not happy.*  *If you want (reinforcer) after the session starts, you can work on (target response).*  *If you stop wanting (reinforcer), get too bored doing (task), or just want to talk to me, you only need to stop (target response) for one minute. You can also just say, ‘I’m done’ or point to this stop card. While you wait, you can (alternative activity) if you like.”* |  |  |  |
| 1. Facilitator ignored participants during the session |  |  |  |
| 1. Facilitator delivered reinforcers according to prescribed schedule requirement throughout the entire assessment |  |  |  |
| 1. Reinforcer morsel size, shape, and quality was approximately the same for all reinforcers delivered. |  |  |  |
| 1. The session terminated either after 1 min of no responding, or after the participant indicated they wanted the session to end. |  |  |  |
| Total |  |  |  |
| Yes / (Yes + No) = |  | | |

**Procedural Fidelity (PFRA Test)**

Date: Session #:

Participant #: Observer initials (prim/reli):

**Schedule Requirement (i.e., FR1, FR4, FR7, etc.):**

| **Procedure** | **Yes** | **No** | **N/A** |
| --- | --- | --- | --- |
| 1. Extraneous materials were removed from assessment area |  |  |  |
| 1. Session termination card and alternative stimulus present. |  |  |  |
| 1. A plate upon which edible morsels of the **target reinforcer** could be placed was available. |  |  |  |
| 1. Facilitator modeled schedule requirement while paraphrasing the following:   *In this version of the game, you can earn ___, by rolling the dice ___ times like this.*  *You can work for as long as you want to earn ____, I’ve bought a lot of them and am happy to keep giving them to you. But it’s important that you only respond for as long as you want ­­­­­____. The moment you’re full, tired of responding, bored, or just want to talk you just need to say you’re done by touching this card. I’ll pay you no matter what, so only respond for as long as you want ____. Can you show me that you know how to say you’re done?*  (if participant is nonverbal, identify valid expressions of dissent) |  |  |  |
| 1. *As needed,* facilitator counted participant responses up through first two reinforcers, then tried to ignore participants for remainder of session. However, responded to participant bids for attention in contextually appropriate ways, with reminders that facilitator should not talk while participant is earning food but that if the participant would prefer to talk, they could end the session. Every five to 10 min, facilitator checks in and asks participants if they would like to stop or if they are still happy earning reinforcers. |  |  |  |
| 1. Reinforcer morsel size, shape, and quality was approximately the same for all reinforcers delivered. |  |  |  |
| 1. The session terminated either after ___ min (1 min recommended, but individualization may be appropriate) of no responding, or after the participant indicated they wanted the session to end. |  |  |  |
| Total |  |  |  |
| Yes / (Yes + No) = |  | | |

**Procedural Fidelity (Extinction Challenge: Baseline)**

Date: Session #:

Participant #: Observer initials (prim/reli):

**Group Assignment:** Cheap AO Cheap EO Expensive AO Expensive EO

**Schedule Requirement** (e.g., FR1, FR4, FR7, etc.):

**Session Duration:**

**Max Reinforcers per Appointment:**

**Max Appointment Duration:**

| **Procedure** | **Yes** | **No** | **N/A** |
| --- | --- | --- | --- |
| 1. Extraneous materials removed from assessment area. |  |  |  |
| 1. Session termination card and alternative stimulus present. |  |  |  |
| 1. A plate upon which edible morsels of the **target reinforcer** could be placed was available. |  |  |  |
| 1. *As needed* (for up to first five sessions of baseline) facilitator modeled schedule requirement while paraphrasing the following:   *In this version of the game, you can earn ___, by rolling the dice ___ times like this.*  *You can work for as long as you want to earn ____, I’ve bought a lot of them and am happy to keep giving them to you. But it’s important that you only respond for as long as you want ­­­­­____. The moment you’re full, tired of responding, bored, or just want to talk you just need to say you’re done by touching this card. I’ll pay you no matter what so only respond for as long as you want ____. Can you should me that you know how to say you’re done?*  (if participant is nonverbal, identify valid expressions of dissent)  For final five sessions, facilitator simply asks “do you remember what to do?” and only provides additional information if requested. |  |  |  |
| 1. Facilitator delivered reinforcers according to prescribed schedule requirement throughout the entire assessment   *As needed (for up to first five sessions of baseline),* facilitator counted participant responses up through first two reinforcers, then tried to ignore participants for remainder of session. However, responded to participant bids for attention in contextually appropriate ways, with reminders that facilitator should not talk while participant is earning food but that if the participant would prefer to talk, they could end the session. |  |  |  |
| 1. Reinforcer morsel size, shape, and quality was approximately the same for all reinforcers delivered. |  |  |  |
| 1. The session terminated at prescribed time |  |  |  |
| 1. Appointment parameters (listed above) were not violated |  |  |  |
| Total |  |  |  |
| Yes / (Yes + No) = |  | | |

**Procedural Fidelity (Extinction Challenge: Extinction)**

Date: Session #:

Participant #: Observer initials (prim/reli):

**Group Assignment:** Cheap AO Cheap EO Expensive AO Expensive EO

**Schedule Requirement** (e.g., FR1, FR4, FR7, etc.):

**Session Duration:**

**Max Reinforcers per Appointment:**

**Max Appointment Duration:**

| **Procedure** | **Yes** | **No** | **N/A** |
| --- | --- | --- | --- |
| 1. Extraneous materials were removed from assessment area. |  |  |  |
| 1. Session termination card and alternative stimulus present. |  |  |  |
| 1. A plate upon which edible morsels of the **target reinforcer** could be placed was available. |  |  |  |
| 1. Facilitator paraphrased the following statement:   *“We are going to start a session, now. This just means I’m going to give you an opportunity to earn (reinforcer).*  *During sessions I cannot talk to you. That’s just a rule I have to follow. It doesn’t mean I don’t want to talk or that I’m not happy.*  *If you want (reinforcer) after the session starts, you can work on (target response).*  *If you stop wanting (reinforcer), get too bored doing (task), or just want to talk to me, you only need to stop (target response) for one minute. You can also just say, ‘I’m done’ or point to this stop card. While you wait, you can (alternative activity) if you like.”* |  |  |  |
| 1. Facilitator completes pre-session task prior to initiating extinction   **AO.** Facilitator sets up area (e.g., target response, alt response, break card) but does not require participant to respond to consume *up to* as many units of reinforcement as the most consumed during any single assessment (check free access, PRA, and PFRA data to gather this information). Once max reinforcers are consumed, or paticpant has indicated they are done. Prompt them to respond until they satisfy the baseline satisfy schedule requirement one time, and then deliver the reinforcement. Afterward, count in and start the session.  **EO.** Facilitator sets up area (e.g., target response, alt response, break card), prompts participant to respond until they satisfy the baseline schedule requirement one time, and then deliver the reinforcement. Afterward, count in and start the session. |  |  |  |
| 1. Facilitator tried to ignore participants for entire session. However, responded to participant bids for attention in contextually appropriate ways, with reminders that facilitator should not talk while participant is earning food but that if the participant would prefer to talk, they could end the session. |  |  |  |
| 1. Facilitator DELIVERED NO REINFORCERS |  |  |  |
| 1. The session terminated at prescribed time, or after participant dissent |  |  |  |
| Total |  |  |  |
| Yes / (Yes + No) = |  | | |
