# Supplementary material for: Evaluating contributions of progressive ratio analysis to economic metrics of demand
Source: J Exp Anal Behav. 2025 Dec 26;125(1):e70077. doi: 10.1002/jeab.70077 (PMC12742636; doi:10.1002/jeab.70077)
Supplement: Supplementary file 2 — Data S2 Supporting Information [file JEAB-125-0-s005.docx]

Table SM-2

*Interobserver Agreement and Procedural Fidelity Scores Across Assessments, By Participant*

|  | **PRA** | | **PFRA** | |
| --- | --- | --- | --- | --- |
| **Participant** | **IOA** | **Fidelity** | **IOA** | **Fidelity** |
| P1 | 99.95 | 100 | 95.70 | 95.83 |
| P2 | 100 | 100 | 100 | 100 |
| P4 | 100 | 100 | 99.66 | 100 |
| P5 | 98.71 | 96.88 | 98.50 | 100 |
| P6 | 100 | 100 | 96.50 | 96.30 |
| P7 | 100 | 100 | 100 | 100 |
| P8 | 98.41 | 97.92 | 99.71 | 100 |
| P9 | 100 | 100 | 100 | 100 |
| P10 | 98.10 | 100 | 94.45 | 100 |
| P11 | 100 | 100 | 100 | 100 |
| P12 | 100 | 100 | 99.67 | 100 |
| P13 | 100 | 100 | 100 | 100 |
| P14 | 100 | 100 | 100 | 100 |
| P15 | 95.59 | 100 | 100 | 100 |
| P16 | 100 | 100 | 100 | 100 |
| P17 | 93.47 | 100 | 100 | 100 |
| P18 | 100 | 100 | 100 | 100 |
| P19 | 98.67 | 100 | 100 | 100 |
| P21 | 100 | 100 | 100 | 100 |
| P22 | 100 | 100 | 100 | 100 |
| P23 | 100 | 100 | 100 | 100 |
| P24 | 100 | 100 | 95.83 | 100 |
| P25 | 99.74 | 100 | 99.91 | 100 |
| P28 | 99.67 | 100 | 97.50 | 100 |
| P29 | 100 | 100 | 100 | 100 |
| P30-B | 98 | 100 | 98.50 | 100 |
| P31 | 100 | 100 | 100 | 100 |
| P32 | 100 | 100 | 100 | 100 |
| P33 | 100 | 100 | 100 | 100 |
| P34 | . | 100 | 100 | 100 |
| P35 | 100 | 100 | 100 | 100 |
| P36 | 100 | 100 | 100 | 100 |
| P37 | 100 | 100 | 99.24 | 100 |
| P38 | 100 | 100 | 100 | 100 |
| P39 | 100 | 100 | 95.54 | 100 |
| P41 | 100 | 100 | 100 | 100 |
| P43 | 100 | 100 | 100 | 100 |
| P44 | 99 | 100 | 100 | 100 |
| P45 | 100 | 95.8 | 100 | 100 |
| P46 | 100 | 100 | 93.50 | 100 |
| P47 | 100 | 100 | 99.50 | 100 |
| P48 | 100 | 100 | 100 | 100 |
| P49 | 100 | 100 | 100 | 100 |
| P50 | 97.5 | 97.92 | 100 | 100 |
| P51 | 100 | 100 | 84.56 | 100 |
| P52-B | 99.29 | 100 | 100 | 100 |
| P53 | 93 | 93.75 | 100 | 100 |
| P54 | 98.5 | 97.92 | 99.50 | 100 |
| P55 | 99.06 | 93.75 | 99.80 | 100 |
| P56 | 99.82 | 95.83 | 99.00 | 100 |
| P57 | 100 | 97.92 | 100 | 100 |
| P58 | 98.22 | 97.92 | 97.60 | 100 |
| P59-B | 95.76 | 100 | 100 | 100 |
| P60-B | 100 | 100 | 100 | 100 |
| P61 | 98.25 | 100 | 99.60 | 100 |
| P62-B | 99.83 | 100 | 99.83 | 100 |
| P63 | 100 | 100 | 100 | 100 |
| P65 | 100 | 100 | 99.70 | 100 |
| P66 | 99.83 | 100 | 100 | 96.43 |
| P67 | 100 | 100 | 100 | 100 |
| P68 | 100 | 100 | 98.06 | 95.23 |
| P69 | 94.44 | 100 | 100 | 100 |
| P70 | 100 | 100 | 100 | 100 |
| P71 | 100 | 100 | . | 100 |
| P72 | 100 | 100 | 99.60 | 100 |
| P73 | 100 | 100 | 98.38 | 100 |
| P74 | 100 | 100 | 100 | 100 |
| P75 | 100 | 100 | 100 | 100 |
| P76 | 100 | 100 | 100 | 100 |
| P77 | 100 | 100 | 90.32 | 100 |
| P78 | 99.15 | 100 | 100 | 100 |
| P79 | 100 | 100 | 100 | 100 |
| P80 | 100 | 100 | 95.45 | 100 |
| P81 | 100 | 100 | 99.23 | 100 |
| P82 | 98.5 | 100 | 94.50 | 100 |
| P83 | 98 | 100 | 100 | 100 |
| P85 | 100 | 100 | 100 | 100 |
| P86 | 100 | 100 | 98.95 | 100 |
| P87 | . | 100 | 100 | 100 |
| P88 | 99.67 | 100 | . | 100 |
| P89 | 96.67 | 100 | 99.80 | 100 |
| P90 | 100 | 100 | 100 | 100 |
| P91 | 100 | 100 | 100 | 100 |
| P92 | 100 | 100 | 100 | 100 |
| P93 | 100 | 100 | 100 | 100 |
| P94 | 100 | 100 | 97.50 | 100 |
| P95 | 100 | 100 | 100 | 100 |
| P96 | 100 | 100 | 100 | 100 |
| M1 | . | 100 | 100 | 100 |
| M2 | . | 100 | 100 | 100 |
| M3 | 100 | 100 | 100 | 100 |
| M4 | . | 100 | 100 | 100 |
| M5 | 100 | 100 | 100 | 100 |
| M6 | 100 | 100 | 100 | 100 |
| M7 | . | 100 | 100 | 100 |
| M8 | . | 100 | 100 | 100 |

*Note.* Scores reflect session averages for each assessment listed. PRA = Progressive-Ratio Reinforcer Analysis; PFRA = Progressive Fixed-Ratio Reinforcer Analysis; IOA = Intorobserver Agreement.
