# Supplementary material for: Evaluating contributions of progressive ratio analysis to economic metrics of demand
Source: J Exp Anal Behav. 2025 Dec 26;125(1):e70077. doi: 10.1002/jeab.70077 (PMC12742636; doi:10.1002/jeab.70077)
Supplement: Supplementary file 3 — Data S3 Supporting Information [file JEAB-125-0-s001.docx]

**Participant Demographic Information and Appointment Details**

Of those participants who completed the study, 55.2% (53 of 96) identified (or were identified) as cisgender men, 42.7% (41 of 96) identified (or were identified) as cisgender women, 1% (1 of 96) identified as a trans-gender man, and 1% (1 of 96) identified as a trans-gender woman. Verbal reports indicated that 88.5% (85 of 96) of participants were White, 8.3% (8 of 96) participants were Black or African American, 2.1% (2 of 96) of participants were Asian (Indian), and 1% (1 of 96) of participants were American Indian/Alaska Native.

The disabilities of 77 participants fell under a single category of the IDEA classification system, the disabilities of 16 participants spanned two IDEA categories, and the diagnoses of 3 participants spanned three IDEA categories. Using the IDEA classification system, 81 participants were reported to have an intellectual disability, 16 participants were autistic, 10 had other health impairments, 3 had orthopedic impairments, 3 had traumatic brain injuries, 2 had speech or language impairments, and 2 had visual impairments.

Table SM-1

*Individual Participant Demographic Information and Appointment Details*

| **Participant** | **Age** | **Race** | **Ethnicity** | **Gender** | **Disability** | **S^R+^** | **Target R** | **1^0^ Appt Setting** |
| --- | --- | --- | --- | --- | --- | --- | --- | --- |
| P1 | 54 | White | Non-Hispanic | Male | ID; VI | Snack | Clothespin | Home |
| P2 | 50 | White | Non-Hispanic | Female | ID | Snack | Dice Roll | Home |
| P4 | 55 | White | Non-Hispanic | Female | ID | Snack | Dice Roll | Home |
| P5 | 57 | Black | Non-Hispanic | Female | ID | Snack | Clothespin | D/W/U |
| P6 | 74 | White | Non-Hispanic | Female | ID | Snack | Dice Roll | D/W/U |
| P7 | 53 | White | Non-Hispanic | Female | ID | Snack | Dice Roll | D/W/U |
| P8 | 58 | White | Non-Hispanic | Male | ID | Snack | Clothespin | D/W/U |
| P9 | 60 | White | Non-Hispanic | Female | ID | Sound | Dice Roll | Home |
| P10 | 78 | White | Non-Hispanic | Male | ID; OI | Snack | Dice Roll | Home |
| P11 | 70 | White | Non-Hispanic | Male | ID; VI | Sound | Dice Roll | Home |
| P12 | 58 | White | Non-Hispanic | Female | ID | Snack | Dice Roll | Home |
| P13 | 30 | White | Non-Hispanic | Female | ID | Snack | Dice Roll | Home |
| P14 | 62 | White | Non-Hispanic | Female | ID | Snack | Dice Roll | Home |
| P15 | 18 | White | Non-Hispanic | Female | ID | Drink | Dice Roll | Home |
| P16 | 43 | White | Non-Hispanic | Male | ID; ASD | Snack | Dice Roll | Home |
| P17 | 48 | Black | Non-Hispanic | Male | ID | Snack | Dice Roll | Home |
| P18 | 36 | White | Non-Hispanic | Male | ID; ASD | Snack | Dice Roll | Home |
| P19 | 74 | White | Non-Hispanic | Female | ID | Snack | Dice Roll | Home |
| P21 | 21 | White | Non-Hispanic | Male | ID; OI | Snack | Dice Roll | D/W/U |
| P22 | 20 | White | Non-Hispanic | Female | ID; SLI | Snack | Dice Roll | Home |
| P23 | 21 | White | Hispanic | Male | ID; ASD; SLI | Snack | Dice Roll | D/W/U |
| P24 | 19 | Black | Non-Hispanic | Female | ID | Snack | Dice Roll | D/W/U |
| P25 | 22 | White | Hispanic | Male | ID | Snack | Dice Roll | D/W/U |
| P28 | 34 | White | Non-Hispanic | Male | OI | Snack | Dice Roll | D/W/U |
| P29 | 31 | White | Non-Hispanic | Male | ID | Snack | Dice Roll | D/W/U |
| P30-B | 22 | Asian (Indian) | Non-Hispanic | Male | ASD | Snack | Dice Roll | D/W/U |
| P31 | 60 | White | Non-Hispanic | Female | ID | Other | Dice Roll | Home |
| P32 | 70 | White | Non-Hispanic | Female | ID | Other | Dice Roll | Home |
| P33 | 57 | White | Non-Hispanic | Female | ID | Other | Dice Roll | Home |
| P34 | 69 | White | Non-Hispanic | Female | ID | Other | Dice Roll | Home |
| P35 | 79 | Black | Non-Hispanic | Female | ID | Other | Dice Roll | D/W/U |
| P36 | 32 | Black | Non-Hispanic | Male | ID | Sound | Dice Roll | D/W/U |
| P37 | 32 | Black | Non-Hispanic | Female | ID | Sound | Dice Roll | D/W/U |
| P38 | 70 | White | Non-Hispanic | Trans-Male | ID | Snack | Dice Roll | D/W/U |
| P39 | 33 | White | Non-Hispanic | Male | ID; VI | Snack | Dice Roll | D/W/U |
| P41 | 19 | White | Non-Hispanic | Female | ID | Snack | Dice Roll | D/W/U |
| P43 | 22 | White | Non-Hispanic | Female | ID | Snack | Dice Roll | D/W/U |
| P44 | 42 | Asian (Indian) | Non-Hispanic | Male | ID | Snack | Dice Roll | D/W/U |
| P45 | 33 | White | Non-Hispanic | Male | ID; ASD | Snack | Dice Roll | Home |
| P46 | 24 | Black | Non-Hispanic | Male | ID; OHI | Snack | Dice Roll | D/W/U |
| P47 | 53 | White | Non-Hispanic | Male | OHI | Snack | Dice Roll | D/W/U |
| P48 | 48 | White | Non-Hispanic | Male | ID; ASD | Snack | Dice Roll | D/W/U |
| P49 | 27 | White | Non-Hispanic | Trans- Female | ID | Snack | Dice Roll | D/W/U |
| P50 | 27 | White | Non-Hispanic | Male | ASD | Snack | Dice Roll | D/W/U |
| P51 | 38 | White | Non-Hispanic | Male | ID | Sound | Dice Roll | D/W/U |
| P52-B | 22 | White | Non-Hispanic | Male | ID | Snack | Dice Roll | D/W/U |
| P53 | 31 | White | Non-Hispanic | Male | ID; OHI | Snack | Dice Roll | D/W/U |
| P54 | 56 | White | Non-Hispanic | Male | ID; OHI | Snack | Dice Roll | D/W/U |
| P55 | 47 | White | Non-Hispanic | Male | TBI | Snack | Dice Roll | D/W/U |
| P56 | 57 | White | Non-Hispanic | Male | OHI | Snack | Dice Roll | D/W/U |
| P57 | 33 | White | Non-Hispanic | Male | ID | Snack | Dice Roll | D/W/U |
| P58 | 34 | White | Non-Hispanic | Male | ID | Sound | Dice Roll | D/W/U |
| P59-B | 29 | White | Non-Hispanic | Female | ID; ASD; OHI | Snack | Dice Roll | D/W/U |
| P60-B | 34 | White | Non-Hispanic | Male | ID | Snack | Dice Roll | D/W/U |
| P61 | 22 | White | Non-Hispanic | Male | ID | Snack | Dice Roll | Home |
| P62-B | 29 | White | Non-Hispanic | Male | ID | Snack | Dice Roll | D/W/U |
| P63 | 51 | White | Non-Hispanic | Male | ID | Snack | Dice Roll | D/W/U |
| P65 | 38 | White | Non-Hispanic | Male | ID | Sound | Dice Roll | D/W/U |
| P66 | 39 | White | Non-Hispanic | Female | ID | Drink | Dice Roll | Home |
| P67 | 58 | White | Non-Hispanic | Male | ID | Drink | Dice Roll | D/W/U |
| P68 | 54 | White | Non-Hispanic | Female | ID | Drink | Dice Roll | Home |
| P69 | 61 | White | Non-Hispanic | Male | ID; ASD | Sound | Dice Roll | D/W/U |
| P70 | 20 | American Indian/Alaska Native | Non-Hispanic | Female | OHI | Snack | Dice Roll | Home |
| P71 | 34 | White | Non-Hispanic | Male | OHI | Snack | Dice Roll | D/W/U |
| P72 | 46 | White | Non-Hispanic | Female | ID | Snack | Dice Roll | D/W/U |
| P73 | 31 | White | Non-Hispanic | Female | OHI | Snack | Dice Roll | Home |
| P74 | 26 | White | Non-Hispanic | Female | ID | Snack | Dice Roll | D/W/U |
| P75 | 25 | White | Non-Hispanic | Male | ID; ASD; OHI | Snack | Dice Roll | Home |
| P76 | 32 | White | Non-Hispanic | Female | ID | Drink | Dice Roll | D/W/U |
| P77 | 55 | White | Non-Hispanic | Male | ID | Snack | Dice Roll | D/W/U |
| P78 | 41 | White | Non-Hispanic | Female | TBI | Snack | Dice Roll | D/W/U |
| P79 | 39 | White | Non-Hispanic | Male | ID; ASD | Snack | Dice Roll | D/W/U |
| P80 | 39 | White | Non-Hispanic | Male | ID | Drink | Dice Roll | D/W/U |
| P81 | 35 | White | Non-Hispanic | Male | ID; ASD | Snack | Dice Roll | D/W/U |
| P82 | 41 | White | Non-Hispanic | Male | ID | Snack | Dice Roll | D/W/U |
| P83 | 34 | White | Non-Hispanic | Male | ID | Snack | Dice Roll | D/W/U |
| P85 | 27 | White | Non-Hispanic | Female | ID | Snack | Dice Roll | D/W/U |
| P86 | 36 | White | Non-Hispanic | Male | ID | Video | Dice Roll | D/W/U |
| P87 | 25 | White | Non-Hispanic | Female | ID | Snack | Dice Roll | D/W/U |
| P88 | 33 | White | Non-Hispanic | Female | ID | Craft | Dice Roll | D/W/U |
| P89 | 25 | White | Non-Hispanic | Male | ID | Snack | Dice Roll | D/W/U |
| P90 | 31 | White | Non-Hispanic | Male | ID | Snack | Dice Roll | D/W/U |
| P91 | 27 | White | Non-Hispanic | Female | ID | Snack | Dice Roll | D/W/U |
| P92 | 34 | White | Non-Hispanic | Female | ID | Snack | Dice Roll | D/W/U |
| P93 | 30 | White | Non-Hispanic | Female | ID | Snack | Dice Roll | D/W/U |
| P94 | 32 | White | Non-Hispanic | Female | ID | Snack | Dice Roll | D/W/U |
| P95 | 26 | White | Non-Hispanic | Female | TBI | Snack | Dice Roll | D/W/U |
| P96 | 23 | Other | Non-Hispanic | Male | ASD | Video | Dice Roll | D/W/U |
| M1 | 61 | White | Non-Hispanic | Male | ASD | Snack | Dice Roll | D/W/U |
| M2 | 31 | White | Non-Hispanic | Male | ASD | Snack | Dice Roll | D/W/U |
| M3 | 37 | White | Non-Hispanic | Female | ID | Snack | Dice Roll | D/W/U |
| M4 | 32 | White | Non-Hispanic | Male | ID | Snack | Dice Roll | D/W/U |
| M5 | 24 | White | Non-Hispanic | Male | ASD | Snack | Dice Roll | D/W/U |
| M6 | 46 | Black | Non-Hispanic | Female | ID | Snack | Dice Roll | D/W/U |
| M7 | 35 | White | Non-Hispanic | Male | ID | Snack | Dice Roll | D/W/U |
| M8 | 25 | White | Non-Hispanic | Female | ID | Snack | Dice Roll | D/W/U |

*Note.* ID = Intellectual Disability; OHI = Other Health Impairment; OI = Orthopedic Impairment; Visual Impairment; SLI = Speech or Language Impairment; TBI = Traumatic Brain Injury; 1^0^ Appt Setting = Primary appointment setting; D/W/U = Day Program/Workshop/University
