# Supplementary material for: Evaluating contributions of progressive ratio analysis to economic metrics of demand
Source: J Exp Anal Behav. 2025 Dec 26;125(1):e70077. doi: 10.1002/jeab.70077 (PMC12742636; doi:10.1002/jeab.70077)
Supplement: Supplementary file 4 — Data S4 Supporting Information [file JEAB-125-0-s003.docx]

**SM Figure 1**

Non-logarithmically transformed relations between basis x BP1, basis x P_max_ and PFRA P_max_.

**SM Figure 2**

Non-logarithmically transformed relations between various basis x PRA metrics and measures of equilibrium obtained from PFRA.

**SM Figure 3**

Participant-specific data obtained from first 18 participants enrolled who completed the study. Demand curves produced by basis x PRA displayed in the unshaded sections of each column. Demand curves produced by PFRA are displayed in the shaded sections of each column.

**Note:** FR = fixed ratio; R = responses emitted; S^R+^ = reinforcers obtained.

**SM Figure 4**

Participant-specific data obtained from the 19^th^ to 36^th^ participants enrolled who completed the study. Demand curves produced by basis x PRA displayed in the unshaded sections of each column. Demand curves produced by PFRA are displayed in the shaded sections of each column.

**Note:** FR = fixed ratio; R = responses emitted; S^R+^ = reinforcers obtained.

**SM Figure 5**

Participant-specific data obtained from the 47^th^ to 54^th^ participants enrolled who completed the study. Demand curves produced by basis x PRA displayed in the unshaded sections of each column. Demand curves produced by PFRA are displayed in the shaded sections of each column.

**Note:** FR = fixed ratio; R = responses emitted; S^R+^ = reinforcers obtained.

**SM Figure 6**

Participant-specific data obtained from the 55^th^ to 72^nd^ participants enrolled who completed the study. Demand curves produced by basis x PRA displayed in the unshaded sections of each column. Demand curves produced by PFRA are displayed in the shaded sections of each column.

**Note:** FR = fixed ratio; R = responses emitted; S^R+^ = reinforcers obtained.

**SM Figure 7**

Participant-specific data obtained from the 73^rd^ to 90^th^ participants enrolled who completed the study. Demand curves produced by basis x PRA displayed in the unshaded sections of each column. Demand curves produced by PFRA are displayed in the shaded sections of each column.

**Note:** FR = fixed ratio; R = responses emitted; S^R+^ = reinforcers obtained.

**SM Figure 8**

Participant-specific data obtained from the 91^st^ to 96^th^ participants enrolled who completed the study. Demand curves produced by basis x PRA displayed in the unshaded sections of each column. Demand curves produced by PFRA are displayed in the shaded sections of each column.

**Note:** FR = fixed ratio; R = responses emitted; S^R+^ = reinforcers obtained.
